# Screening of Galician grapevine varieties by SNPs, phenotypic traits, and phytopathology

**DOI:** 10.3389/fpls.2024.1359506

**Published:** 2024-02-16

**Authors:** Ángela Díaz-Fernández, M. Dolores Loureiro, Santiago Pereira-Lorenzo, Javier Ibáñez, Emilia Díaz-Losada

**Affiliations:** ^1^ Department of Viticulture, Estación de Viticultura e Enoloxía de Galicia (EVEGA)‐Axencia Galega da Calidade Alimentaria (AGACAL), Leiro, Ourense, Spain; ^2^ Department of Crop Production and Engineering Projects, Santiago de Compostela University (USC), Lugo, Spain; ^3^ Department of Viticulture, Instituto de Ciencias de la Vid y del Vino (ICVV), Consejo Superior de Investigaciones Científicas (CSIC), Universidad de la Rioja (UR), Gobierno de la Rioja, Logroño, Spain

**Keywords:** grapevine varietal diversity, genetic structure, ampelography, berry quality, downy mildew, phenology, SNP

## Abstract

The genetic erosion of the European grapevine diversity in the last century has promoted the conservation of varieties in germplasm banks to prevent their disappearance. The study of these varieties is necessary as it would allow the diversification of the wine market, as well as provide a source of genes to face new pathogens or climate constraints. In this work, the grapevine varieties preserved in the “Estación de Viticultura e Enoloxía de Galicia” (EVEGA) Germplasm Bank (Ourense, Spain) were widely characterized, combining ampelography, ampelometry, agronomy, and phytopathology. Moreover, genetic characterization was carried out through the analysis of 48 single-nucleotide polymorphisms (SNPs). A Bayesian analysis based on the SNP data was carried out to define the genetic structure of the EVEGA Germplasm Bank, which allowed the differentiation of two main reconstructed panmictic populations (RPPs), confirming previous results obtained based on microsatellite markers (SSRs). A great diversity between varieties was found for almost every parameter evaluated for ampelography, ampelometry, phytopatology, phenology, and berry quality. A principal component analysis (PCA) performed with these phenotypical data allowed discrimination among some groups of varieties included in different genetic populations. This study allowed us to evaluate the grapevine diversity maintained in the EVEGA Germplasm Bank and characterize varieties of potential value for breeding programs of interest for the Galician viticulture.

## 1 Introduction

The genetic erosion of European grapevine diversity started in the second half of the 19th century, with a great negative impact caused by the phylloxera [*Daktulosphaira vitifoliae* (Fitch)] (García et al., 2020), as well as with the arrival of two other important diseases, powdery [*Erysiphe necator* (Burr.)] and downy mildews [*Plasmopara viticola* (Berk. & Curtis) Berl. & de Toni] from the American continent (Hesler, 2008), with downy mildew being considered as one of the most destructive grapevine diseases in those areas that have a humid and warm climate (Gaforio et al., 2015). The impoverishment of the varietal diversity got worse in the 20th century because of the homogenization of the wine market and the limitations imposed in quality schemes such as Protected Designations of Origin (PDO) or Protected Geographical Indications (PGI), which have strict production practices, including a limited number of grapevine varieties authorized to be grown and that can be used for their wine elaborations (Candiago et al., 2022). In recent years, the European Union’s vineyard reconversion and restructuring policies have caused the loss of old vineyards, where a high diversity was maintained. In terms of preventing the disappearance of further grapevine diversity, prospecting work and conservation of grapevine varieties in regional, national, and international germplasm banks have been developed for a long time in many viticultural regions worldwide (Martín et al., 2011; Augusto et al., 2021). The *Vitis* International Variety Catalogue (*V*IVC database, https://www.vivc.de/) compiles a complete and constantly updated information database about the *Vitis* genotypes existing in 135 grapevine collections from 45 countries worldwide, seven of them from Spain. At present (December 2023), the *V*IVC database has information on more than 13,000 varieties of *Vitis vinifera* L. subsp. *vinifera*, of which approximately 2,000 are registered for wine elaboration in Europe, although only a few hundred varieties are in fact cultivated for this purpose. Databases such as *V*IVC facilitate the exchange of information, including molecular and morphological data, allowing varietal identification and the establishment of synonyms and homonyms, as well as the inference of the genetic structure, diversity, and varietal parentage relationships (Lacombe et al., 2013; Cunha et al., 2016; Duchêne, 2016; Cunha et al., 2020).

Long years of oblivion of the minority varieties that are now conserved in germplasm banks have led to a total or a partial lack of knowledge about their origin, their agronomic and oenological performance, and even their identity. Initially, the identification of the grapevine germplasm diversity in the collections was carried out morphologically by ampelography; nevertheless, this methodology is limited by drawbacks such as the environmental influence on some morphological characteristics, the similarity between some varieties that makes their differentiation difficult, the presence of clones within varieties with differences between their phenotypes, the lack of expert ampelographers, and the time required to provide results, given that plants have to be adult and the studies have to be performed for at least 2 years (This et al., 2004). To overcome these drawbacks, genetic techniques based on DNA methodologies were developed; at first, microsatellite markers (SSRs) have mainly allowed for much faster and more reliable identification and the possibility of establishing synonyms and homonyms among apparently different varieties, proving to be an essential tool to manage grapevine collections. However, ampelography is also being carried out as it provides important information for characterization, breeding programs, and conservation purposes (Bounab and Laiadi, 2019; Dallakyan et al., 2015; Khalil et al., 2017; Cretazzo et al., 2022). Recently, the single-nucleotide polymorphism (SNP) markers have been incorporated into the evaluation of the genetic diversity and the varietal identification because of their abundance in the genome, their high reproducibility, and the possibility of high-throughput detection (Cabezas et al., 2011). These markers allow one to study the genetic diversity and analyze complex traits of utility for breeding programs (Laucou et al., 2018; Augusto et al., 2021; D’Onofrio et al., 2021).

In addition, the acquisition of an extensive knowledge about the agronomical and oenological performance of these varieties is also essential as it provides the basis for choosing the most suitable ones to be planted in a specific location, which is of utmost importance in the current context of climate change. Studies performed in recent years with some of these minority varieties have allowed researchers to know them in depth and evaluate their oenological potential, among other characteristics, like, for instance, with “Brancellao”, “Mencía”, “Merenzao”, “Castañal”,”Albariño”, or “Loureira” for new plantings, being successfully introduced in some PDOs and filling a market niche with different and high-quality wines, linked to the concept of terroir, which surprise consumers and wine professionals (Letaief et al., 2007; Vilanova and Martínez, 2007; Álvarez et al., 2011; Cortés and Díaz, 2011; Vilanova and Freire, 2017).

The success of Galician wines (Northwestern Spain) is largely due to its clear commitment with its exclusive varieties, many of which were nearly extinct until a few years ago (Cortés and Díaz, 2011). The “Estación de Viticultura e Enoloxía de Galicia” (EVEGA, Ourense, Spain) is the institutional entity in charge of preserving all the grapevine diversity found in the vineyards of the Autonomous Community of Galicia. The varieties and hybrids preserved in its Grapevine Germplasm Bank were recovered from old vineyards identified through extensive prospecting works carried out from the 1980s onwards throughout the Galician region.

The unequivocal identification of the grapevine varieties located in the EVEGA Germplasm Bank, as well as their genetic structure, have been established by SSRs in previous studies (Díaz-Losada et al., 2012; Díaz-Losada et al., 2013a). Some of these varieties have also been recently characterized by their aromatic (Díaz-Fernández et al., 2022a; Díaz-Fernández et al., 2022b) and phenolic (Díaz-Fernández et al., 2022c; Díaz-Fernández et al., 2023) profiles. The results of these extensive characterization studies have led to the discovery of previously unidentified varieties in some cases, as has recently occurred with “Albilla do Avia”, previously studied and referred to as “Albilla” (Díaz-Losada et al., 2013b), and to its inscription in the Spanish National Catalogue.

Considering the above issues, the main aim of this study was to perform an extended analysis and complete the characterization of the existing diversity in the EVEGA Grapevine Germplasm Bank looking to evaluate its potential for breeding programs and to diversify the present production. In order to achieve this, a multidisciplinary study that combines ampelography, ampelometry, agronomy, phytopathology, and genetic traits has been carried out. Possible relationships between the genetic traits and the other parameters studied have also been evaluated. Results obtained have allowed researchers to identify interesting varieties in terms of phenology or berry quality. Furthermore, other traits, such as a lower susceptibility to *P. viticola* by some varieties, could help to achieve improvements towards an economic and environment sustainability in viticulture.

## 2 Materials and methods

### 2.1 Plant material and site description

Plants from 82 grapevine accessions located in the EVEGA Grapevine Germplasm Bank were included in the study. The experimental vineyard is situated in the Northwest Spain (Ourense, Galicia -42° 21′ 34.5′′ N 8° 07′08.2′′ W, elevation 87 MAMSL). The vineyard has a surface area of 8,600 m^2^ and an east–west orientation, and it is established in a granitic soil, with a sandy loam texture, a pH (H_2_O) of 6.0, 2.9% of organic matter, 63 ppm of available phosphorus, 278 ppm of assimilable potassium, 154 ppm of exchangeable magnesium, and a cation exchange capacity of 8.11 cmol (+)·kg^−1^. The climate of this region is classified as IH-1 IS-1 FN+2 following the Multicriteria Climatic Classification System (MCC) (Tonietto and Carbonneau, 2004), that is, temperate and sub-humid climate with very cool nights (Blanco, 2008). Vines are approximately 30 years old. They are all grafted on 196-17 C rootstock, with a planting frame of 1.2 × 1.8 m, and trained into a vertical trellis system (VSP) in a double Royat Cordon. Accessions are in plots of 6 to 11 vines each one.

The list of accessions included in this study, their prime *V*IVC (*V*IVC database, https://www.vivc.de/) name and code, and different synonyms are detailed in **Table 1**. A total of 53 of them were phenotypically studied.

**Table 1:** List of accessions studied, their corresponding VIVC (*V*IVC database, https://www.vivc.de/) prime name and codes, local synonyms, and origin of the 48 SNP data used in this work.

Variety (EVEGA collection name)	Prime name *V*IVC	*V*IVC code	Synonyms	Origin of SNP data
“Agudelo”	“Chenin Blanc”	*V*IVC 2527		ICVV-DNA
“Albarín Tinto”	“Alfrocheiro”	*V*IVC 277	“Caíño Gordo”, “Tinto Serodo”	Cunha et al., 2016
“Albariño”	“Alvarinho”	*V*IVC 15689		Cunha et al., 2016
“Albilla do Avia”	“Albilla”	*V*IVC 24392	“Albillo”	This work
“Aleatico”	“Aleatico”	*V*IVC 259		ICVV-DNA
“Aramon”	“Aramon”	*V*IVC 544		ICVV-DNA
“Arinto”	“Arinto”	*V*IVC 602		ICVV-DNA
“Batoca”	“Batoca”	*V*IVC 1037	“Alvacara”, “Treixadura Francesa”	Cunha et al., 2016
“Blanca de Galicia”		nc		This work
“Blanca de Monterrei”	“Carrega Blanco”	*V*IVC 2124	“Branca de Monterrei”	This work
“Blanca de Ribeiras”		nc		This work
“Brancellao”	“Alvarelhao”	*V*IVC 1650	“Albarello”, “Brancello”, “Brencello”, “Serradelo”	Cunha et al., 2016
“Brancellao Blanco”	“Brancellao Blanco”	*V*IVC 24129	“Brancellao Branco”	ICVV-DNA
“Branco Lexítimo”	“Albarín Blanco”	*V*IVC 22838	“Blanco Lexítimo”, “Branca do País”, “Branca Lexítima”, “Blanca Lexítima”, “Raposo”	ICVV-DNA
“Caíño Blanco”	“Caiño Blanco”	*V*IVC 371	“Caíño Branco”	ICVV-DNA
“Caíño Bravo”	“Amaral”	*V*IVC 818	“Caíño Astureses”	Cunha et al., 2016
“Caíño Longo 1”	“Caíño Longo 1”	*V*IVC 5178		This work
“Caíño Longo 2”	“Caíño Longo 2”	*V*IVC 24614		This work
“Caíño Tinto”	“Borraçal”	*V*IVC 1564	“Cachón”, “Cachiño”, “Caíño Redondo 1”, “Tinta Femia 2”, “Tinto Redondo”	Cunha et al., 2016
“Carrasquín”	“Carrasquín”	*V*IVC 2123		ICVV-DNA
“Castañal”	“Castañal”	*V*IVC 23051		Cunha et al., 2016
“Catalán”	“Catawba”	*V*IVC 2346		This work
“Corbillón”	“Docal Tinto”	*V*IVC 3612	“Cascón”	Cunha et al., 2016
“Cuatendrá”		*V*IVC 22846		ICVV-DNA
“Cruce Paco”		nc		This work
“Dona Branca”	“Siria”	*V*IVC 2742	“Moza Fresca”, “Valenciana”	This work
“Espadeiro”	“Camaraou Noir”	*V*IVC 2017	“Couxo”, “Caíño Redondo”	ICVV-DNA
“EVEGA 3”		nc		This work
“EVEGA 4”		nc		This work
“EVEGA 5”	“Náparo”	*V*IVC 8345		This work
“EVEGA 6”	“Doce”	*V*IVC 17674		This work
“Fernąo Pires”	“Fernąo Pires”	*V*IVC 4100		Cunha et al., 2016
“Ferrón”	“Manseng Noir”	*V*IVC 7340		Cunha et al., 2016
“Garnacha”	“Garnacha Roja”	*V*IVC 4980	“Garnacha Roya”	ICVV-DNA
“Garrido Fino”	“Garrido Fino”	*V*IVC 4470		ICVV-DNA
“Gewürztraminer”	“Gewürztraminer”	*V*IVC 12609	“Traminer”	ICVV-DNA
“Godello”	“Gouveio”	*V*IVC 12953	“Cumbrao”	Cunha et al., 2016
“Gold”	“Gold”	*V*IVC 4997		ICVV-DNA
“Gran Negro”	“Grand Noir”	*V*IVC 5012	“Grand Noir de la Calmette”, “Negrón”	ICVV-DNA
“Híbrido”		nc		
“Italia”	“Italia”	*V*IVC 5582		Cabezas et al., 2011
“Jarrosuelto”	“Jarrosuelto”	*V*IVC 24138		ICVV-DNA
“Lado”	“Lado”	*V*IVC 23156		ICVV-DNA
“Loureira”	“Loureiro Blanco”	*V*IVC 6912	“Loureiro Branco”, “Marqués”	Cunha et al., 2016
“Malvasía Bianca”	“Malvasía Moscata”	*V*IVC 22748		This work
“Mandón”	“Garro”	*V*IVC 7326		ICVV-DNA
“Mencía”	“Mencía”	*V*IVC 7623		Cunha et al., 2016
“Merenzao”	“Trousseau Noir”	*V*IVC 12668	“Bastardo”, “Carnaz”, “María Ordoña”, “Pecho”, “Roibal”	Cunha et al., 2016
“Moravia Dulce”	“Marufo”	*V*IVC 8086		Cunha et al., 2016
“Moscatel de Alejandría”	“Muscat of Alexandría”	*V*IVC 8241		Cabezas et al., 2011; Zinelabidine et al., 2014
“Moscatel de Bago Miúdo”	“Muscat a petit grains blancs”	*V*IVC 8193	“Moscatel Galego”	Cunha et al., 2016
“Moscatel de Hamburgo”	“Muscat Hamburg”	*V*IVC 8226		ICVV-DNA
“Moscatel Rubio”	“Mencía”	*V*IVC 7623		This work
“Mosteiro 14”	“Trajadura”	*V*IVC 12629		This work
“Mouratón”	“Mouratón”	*V*IVC 8082	“Mencía Gorda”, “Negreda”	Cunha et al., 2016
“Náparo”	“Náparo”	*V*IVC 8345		ICVV-DNA
“Negrón de Aldán 1”	“Mouratón”	*V*IVC 8082		This work
“Olho de Pargo”	“Gonçalo Pires”	*V*IVC 4891		This work
“Ollo de Sapo”	“Ratiño”	*V*IVC 24127		This work
“Palomino”	“Palomino Fino”	*V*IVC 8888	“Jerez”, “Xerez”	Cabezas et al., 2011; Cunha et al., 2016
“Pan y Carne”	“Estaladiña”	*V*IVC 26281		ICVV-DNA
“Pedral”	“Pedral”	*V*IVC 9078	“Pedrol”	This work
“Picapoll Negro”	“Piquepoul Noir”	*V*IVC 9298		ICVV-DNA
“Pinot”	“Pinot Noir”	*V*IVC 9279		ICVV-DNA
“Pirixileira”	“Chasselas Cioutat”	*V*IVC 2476		ICVV-DNA
“Planta Fina”	“Planta Fina”	*V*IVC 9542		Zinelabidine et al., 2014; Cunha et al., 2016
“Prieto Picudo”	“Prieto Picudo Tinto”	*V*IVC 9694		ICVV-DNA
“Promisión”	“Nehelescol”	*V*IVC 8467		Cunha et al., 2016
“Ratiño”	“Ratiño”	*V*IVC 24127	“Cajarrento”	This work
“Silveiriña”	“Folgasao”	*V*IVC 4178		Cunha et al., 2016
“Sousón”	“Vinhao”	*V*IVC 13100	“Pazao”, “Retinto”, “Tintilla”, “Viñón”	ICVV-DNA
“Syrah”	“Syrah”	*V*IVC 11748		ICVV-DNA
“Tempranillo”	“Tempranillo Tinto”	*V*IVC 12350	“Arauxa”	Cabezas et al., 2011; Cunha et al., 2016
“Tinta de Bares”		nc		This work
“Tinta da Zorra”	“Bouschet Petit”	VIVC 1619	“Tinta da Zorra”	ICVV-DNA
“Torrontés”	“Malvasía Fina”	*V*IVC 715		Cunha et al., 2016
“Treixadura”	“Trajadura”	*V*IVC 12629		Cunha et al., 2016
“Treixadura José Hermo”		nc		This work
“Verdello Blanco”		nc		This work
“Verdello Sebio”	“Verdejo Blanco”	12949		This work
“Xafardán”		nc	“Tinta Oubiña”, “Albariño tinto”	This work
“Zamarrica”	“Cainho da Terra”	*V*IVC 26692	“Caíño da Terra”, “Tinta Femia 1”	This work

nc: no VIVC code (on 12 December 2023).

### 2.2 Measured parameters

#### 2.2.1 SNP analysis

Out of the 82 accessions included in the study, 27 were fully genotyped for 48 SNPs. For the remaining 55 accessions included in this study, there was a previous genetic identification through microsatellite markers (SSRs) (Díaz-Losada et al., 2012; Díaz-Losada et al., 2013a). This information allowed us to directly assign the existing SSR genotype values with the corresponding SNP profiles already stated by the “Instituto de Ciencias de la Vid y el Vino” (ICVV) DNA database (Cabezas et al., 2011; Cunha et al., 2016; ICVV-DNA database (data non-published); Zinelabidine et al., 2014). SNP data origin for each accession is shown in the last column of **Table 1**.

Genotyping was developed as follows: First of all, DNA was extracted from young leaves using the DNeasy Plant kit (Qiagen, Hilden, Germany). Afterwards, NanoDrop 2000 C UV-Vis spectrophotometer (Thermo Scientific, Waltham, MA, USA) was used to check the quality and quantify the DNA concentration, adjusting final DNA concentrations to 5 ng·μL^−1^.

SNP analysis was done using the 48 SNPs proposed by Cabezas et al. (2011). SNP genotyping was carried out as described by Augusto et al. (2021), through the Fluidigm (San Francisco, CA, USA) technology. Genotyping services were provided by the Sequencing and Genotyping Unit of the University of the Basque Country. SNP profiles obtained for the 48 SNPs were pairwise compared with those of the ICVV-SNP database for varietal identification.

#### 2.2.2 Ampelographic and ampelometric characterization

Ampelographic characterization was carried out during three seasons (2014–2016) by two ampelographers, following the “OIV descriptor list for grape varieties and *Vitis* species” second edition methodology (OIV, 2007). A total of 57 ampelographic descriptors in young shoot, shoot, young leaf, mature leaf, flower, bunch, and berry were recorded (**Table 2**). A model description was developed for each variety by selecting the mode of the values obtained for each descriptor. In addition, 17 ampelometric descriptors were measured in herborized mature leaf using the ImageJ software (Image Processing and Analysis in Java, https://imagej.nih.gov/ij/) (**Table 2**), and data were transposed into a qualitative notation following the methodology of the OIV (2007).

**Table 2:** Measured ampelographic and ampelometric descriptors.

Descriptors	Organ	OIV code
Ampelographic	Young shoot	OIV 001, OIV 002, OIV 003, OIV 004, OIV 005
	Shoot	OIV 006, OIV 007, OIV 008, OIV 009, OIV 010, OIV 011, OIV 012, OIV 015-1, OIV 015-2
	Young leaf	OIV 051, OIV 053, OIV 056
	Mature leaf	OIV 065, OIV 067, OIV 068, OIV 069, OIV 070, OIV 071, OIV 072, OIV 073, OIV 074, OIV 075, OIV 076, OIV 078, OIV 079, OIV 080, OIV 081-1, OIV 081-2, OIV 082, OIV 083-1, OIV 083-2, OIV 084, OIV 085, OIV 086, OIV 087, OIV 088, OIV 089, OIV 090, OIV 091, OIV 093
	Flower	OIV 151
	Bunch	OIV 202, OIV 204, OIV 206, OIV 207, OIV 208, OIV 209
	Berry	OIV 220, OIV 221, OIV 222, OIV 223, OIV 225
Ampelometric	Mature leaf	OIV 601 to OIV 617

#### 2.2.3 Phytopathological traits: susceptibility degree to *Plasmopara viticola* through leaf disc test

- Fungal material. A pure culture of *P. viticola* (downy mildew) was isolated from naturally infected “Albariño” plants from an experimental vineyard of EVEGA, located in Ribadumia (Pontevedra, Spain), that did not have any fungicide treatments. A suspension of sporangia was sprayed on “Mencía” leaves and maintained in a chamber in plates at 25°C to obtain the inoculum for the laboratory testing. Then, infected “Mencía” leaves were soaked in sterile distilled water to prepare the sporangia suspension (25,000 sporangia mL^−1^).

- Plant material. Ten two-bud cuttings were taken in January 2016 from each variety and left to sprout in peat in a chamber under controlled conditions at 23 ± 2°C, 60 ± 2% of relative air humidity and a photoperiod of 16/8 h (light/dark, respectively).

- Degree of susceptibility determined by leaf disc test. Leaves (5th to 6th position on the shoot) from the plants grown in the chamber were surface sterilized with 75% ethanol, rinsed with distilled water, and then dried with filter paper. For each variety, 33 discs of 16 mm diameter were punched out of the leaves with a cork borer and placed bottom side up in Petri dishes with humid filter paper. Three replicates of 10 leaf discs were inoculated for each variety. An additional disc in each repetition was mock inoculated, acting as a control. Discs were inoculated with a 50-µL droplet of the *P. viticola* inoculum suspension (25,000 sporangia mL^−1^) and maintained at 24°C with a relative humidity >95% in the dark for 24 h. Then, the plates were subjected to a photoperiod of 16/8 h (light/dark, respectively) for 6 days.

- Measured parameters. Disease incidence, sporulation density, and disease severity were evaluated. Disease incidence was expressed as the percentage of discs showing sporulation or necrosis in relation to the total number of inoculated discs. The sporulation density was qualitatively scored using the “Sporulation density scale” (0—No sporangia; 1—Few sporangia; 2—Moderate presence, sporangia in different groups; 3—High presence of sporangia; 4—Very high presence of sporangia). Disease severity (sporulation area) was assessed using the free software ImageJ (Image Processing and Analysis in Java, https://imagej.nih.gov/ij/) to measure the percentage surface area of each disc with symptoms of sporulation; the data obtained were transferred to a qualitative scale according to the following values: 0 (0% percentage of occupied area); 1 (>0%–25.0%); 2 (>25.0%–50.0%); 3 (>50.0%–75.0%); 4 (>75.0%).

#### 2.2.4 Phenological stages

Baggiolini phenological scale (Baggiolini, 1952) was used to record data of budburst (C), flowering (I), and veraison (M) stages for each variety, expressed in number of days after March 1. Harvest date (N) was established through periodical maturation controls. Growing Degree Days (GDDs) for every phenological stage were also calculated by averaging the daily maximum and minimum temperatures and subtracting the base temperature for grapevine (10°C). The budburst to harvest period and the flowering–veraison and veraison–harvest periods were determined. Data were recorded for 3 years.

#### 2.2.5 Quality parameters of the berry

Must analysis of the different varieties were carried out to evaluate the quality parameters of their berries.

Approximately 500 berries were manually harvested from different parts of the clusters to obtain a representative sample, establishing the harvest date for each variety according to the results obtained in weekly controls monitoring the sugar content, pH, acidity, and sanitary conditions of grapes from veraison to the harvest data. The main aim was to collect the grapes in their better ripening stage (20–23° Brix) depending on the grape variety and their sanitary condition. Samples were crushed with a motorized grape crusher and the following physicochemical parameters were assessed: Total soluble solids (°Brix), pH, and titratable acidity (g tartaric acid·L^−1^) were determined by Fourier transform infrared spectrometry (FTIR) (OENOFOSS™, FOSS, Denmark). Malic and tartaric acids (g·L^−1^) were measured with a LISA 2000 chemical autoanalyzer (HYCEL DIAGNOSTICS, Germany), calibrated following the official methods (OIV, 2009). Samples were taken for a minimum of 2 years.

### 2.3 Data analysis

Statistical differences among the mean values for the different quantitative data related to downy mildew (*P. viticola*) susceptibility degree, phenology, and berry quality were analyzed using one-way analysis of variance (ANOVA). Means were compared with Tukey’s test. XLstat‐Basic+ (Addinsoft, Paris, France) software was used for previous analysis. Qualitative data obtained from the ampelographic and ampelometric evaluation, together with downy mildew (*P. viticola*) evaluation data in leaf discs, sporulation density, and disease severity (previously transformed to a qualitative scale), were subjected to a principal component analysis (PCA). This same analysis was also computed with phenology and berry quality quantitative data, by considering the genetic structure based on SNP obtained in this study, and the one based on SSRs obtained by Díaz-Losada et al. (2012). PCAs were performed with the SPSS statistics SPSS V.28 (IBM, Armonk, NY, USA) software.

Genetic structure was studied by a Bayesian method performed with the Structure software (Pritchard et al., 2000a; Pritchard et al., 2000b) by using the admixture model with unlinked loci and correlated allele frequencies, as defined by Porras-Hurtado et al. (2013), who recommended over 20 iterations (30 in this study) to estimate the ancestry membership proportions of a population. *K* = 1 to 15 unknown reconstructed panmictic populations (RPPs) of genotypes were computed, with the option to use popinfo = 0, popflag = 0, which consider that the sampled genotypes were of unidentified origin, assigning them probabilistically to RPPs based on a qI (probability of membership) of 80%. In this study, a threshold of 80% was used, as previously used in other studies such as Díaz-Losada et al. (2012), including those with a lower than 80% probability in an admixed group. An average of the 30 iterations carried out has been used for the graphical results displayed. The second-order change of the likelihood function, divided by the SD of the likelihood (Δ*K*), was also estimated to find the best *K* value supported by the data (Evanno et al., 2005) using Structure Harvester (Earl and von Holdt, 2012).

## 3 Results

### 3.1 Genetic diversity by SNPs

The 82 accessions included in this study corresponded to 76 different varieties. SNP genotyping worked properly, and in 26 of the 27 samples, at least 43 SNP loci could be genotyped (**Supplementary Table S1**). SNP analysis allowed us to establish the identity of 15 of the 27 accessions under study, corresponding in several cases to existing *V*IVC (*V*IVC database, https://www.vivc.de/) prime names (**Table 1**): “Gonçalo Pires” (*V*IVC 4891), “Pedral” (*V*IVC 9078), and “Ratiño” (*V*IVC 24127), and with known synonyms: “Zamarrica” (*V*IVC 26692, “CAINHO DA TERRA”), “Blanca de Monterrei” (*V*IVC 2124, “CARREGA BRANCO”), “Catalán” (*V*IVC 2346, “CATAWBA”), “Malvasia Bianca” (*V*IVC 22748, “MALVASIA MOSCATA”), and “Dona Branca” (*V*IVC 2742, “SIRIA”). “Negrón de Aldán 1” was erroneously named as it matched “Mouratón” (*V*IVC 8082, “MOURATON”). “Verdello Sebio” matched *V*IVC 12949, “VERDEJO BLANCO”, but it cannot be considered a new synonym. Several unnamed samples were identified: “EVEGA 5” corresponded to *V*IVC 8345 “NAPARO”, “EVEGA 6” corresponded to *V*IVC 17674 “DOCE”, and “Mosteiro 14” matched *V*IVC 12629 “TRAJADURA”. Some mistakes could also be identified in the Collection: “Ollo de Sapo”, paired with “Ratiño” (*V*IVC 24127 “RATINO”), while it really corresponds to *V*IVC 1564 “BORRAÇAL”. The accession wrongly named “Moscatel Rubio”, which really pairs with “Mencía” (*V*IVC 7623, “MENCIA”). SNP genotypes obtained for the remaining 12 accessions were unique or only matched with other Galician samples previously studied in the ICVV-DNA database: “Albilla do Avia”, “Blanca de Ribeiras”, “Blanca de Galicia”, “Caíño Longo 1”, “Caíño Longo 2”, “Cruce Paco”, “EVEGA 3”, “EVEGA 4”, “Tinta de Bares”, “Treixadura José Hermo”, “Xafardán”, and “Verdello Blanco”. SNP analysis also confirmed that “Xafardán” is not an “Albariño” somatic variant as it had been hypothesized since it was also referred to as “Albariño Tinto”.

### 3.2 Genetic and geographic structure

**Figure 1:**
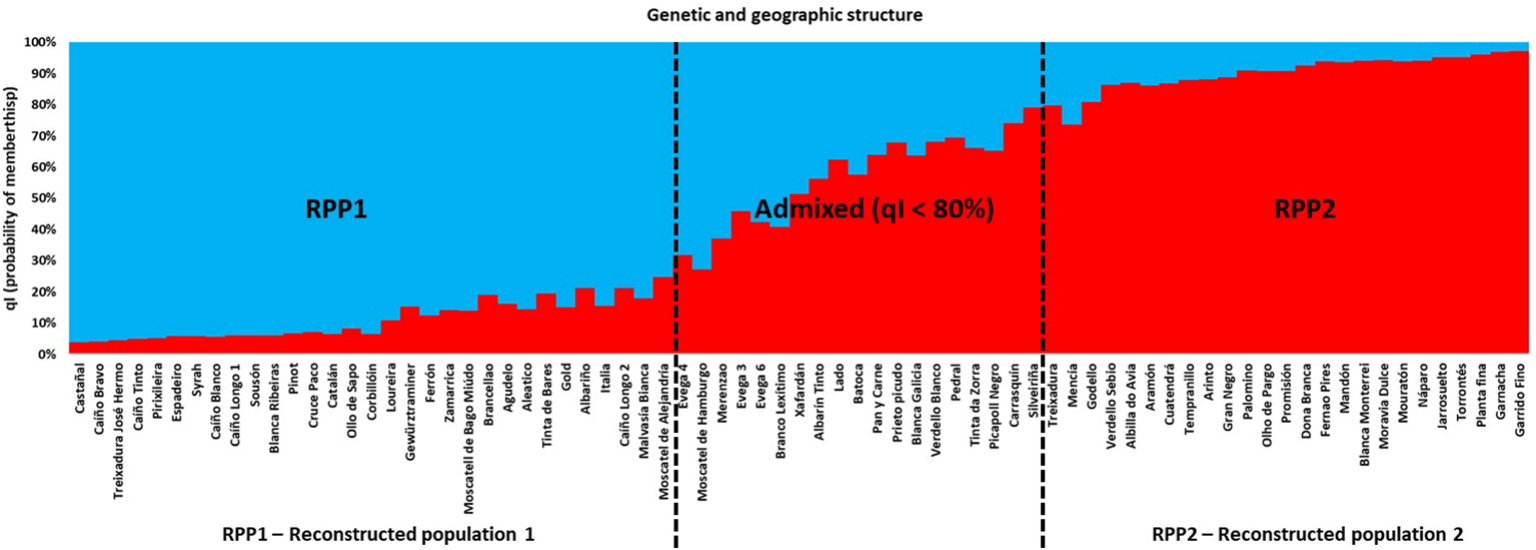
Bayesian analysis for the reconstructed panmictic populations (RPP) obtained via Structure based on data for 48 SNPs in the different genotypes.

A Bayesian analysis, using the Structure software (Porras-Hurtado et al., 2013), was conducted using 48 SNPs to determine the genetic structure among the unique genotypes. *K* = 2 (**Supplementary Figure S1**) was the most likely estimation according to the Δ*K* criterion by using Structure Harvester (Earl and Von Holdt, 2012) in a group of 56 genotypes out of 75, with a qI (probability of membership) > 80% (75% of all genotypes), which corresponded to a strong differentiation in two main groups of genotypes (RPP). One included 31 genotypes (RPP1, 41% of the total number of genotypes, **Figure 1**), all of them from Western Galician, unless for “Moscatel de Bago Miúdo” (“Moscatel Morisco”). A second one grouped 25 genotypes (RPP2, 33% of the total number of genotypes), mainly varieties of other Spanish regions, but also Galician varieties. It was considered that the sampled genotypes were of unidentified origin (admixed group), when assigning them probabilistically to RPPs based on a qI < 80%.

### 3.3 Ampelographic and ampelometric parameters: susceptibility degree to *Plasmopara viticola*

**Figure 2:**
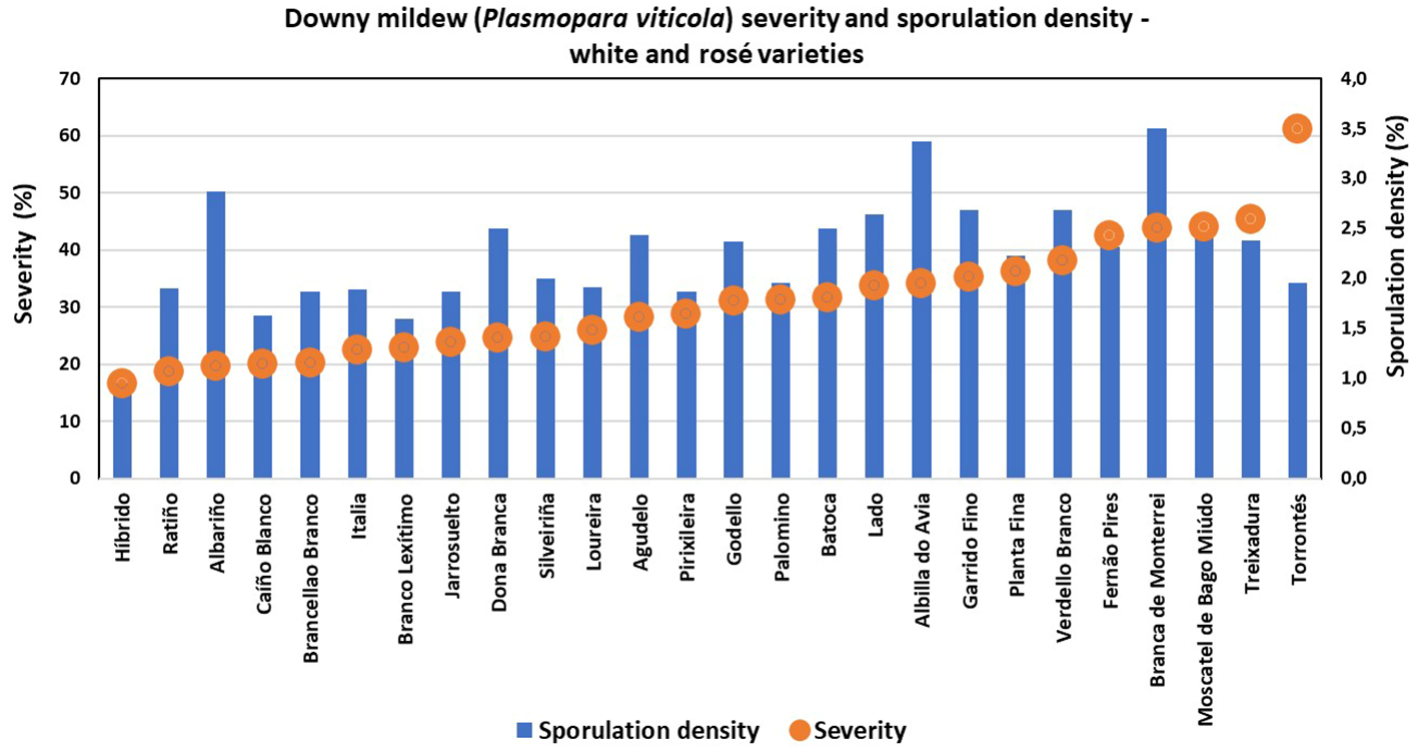
Degree of susceptibility to downy mildew (*Plasmopara viticola*) of different white and rosé grape varieties.

**Figure 3:**
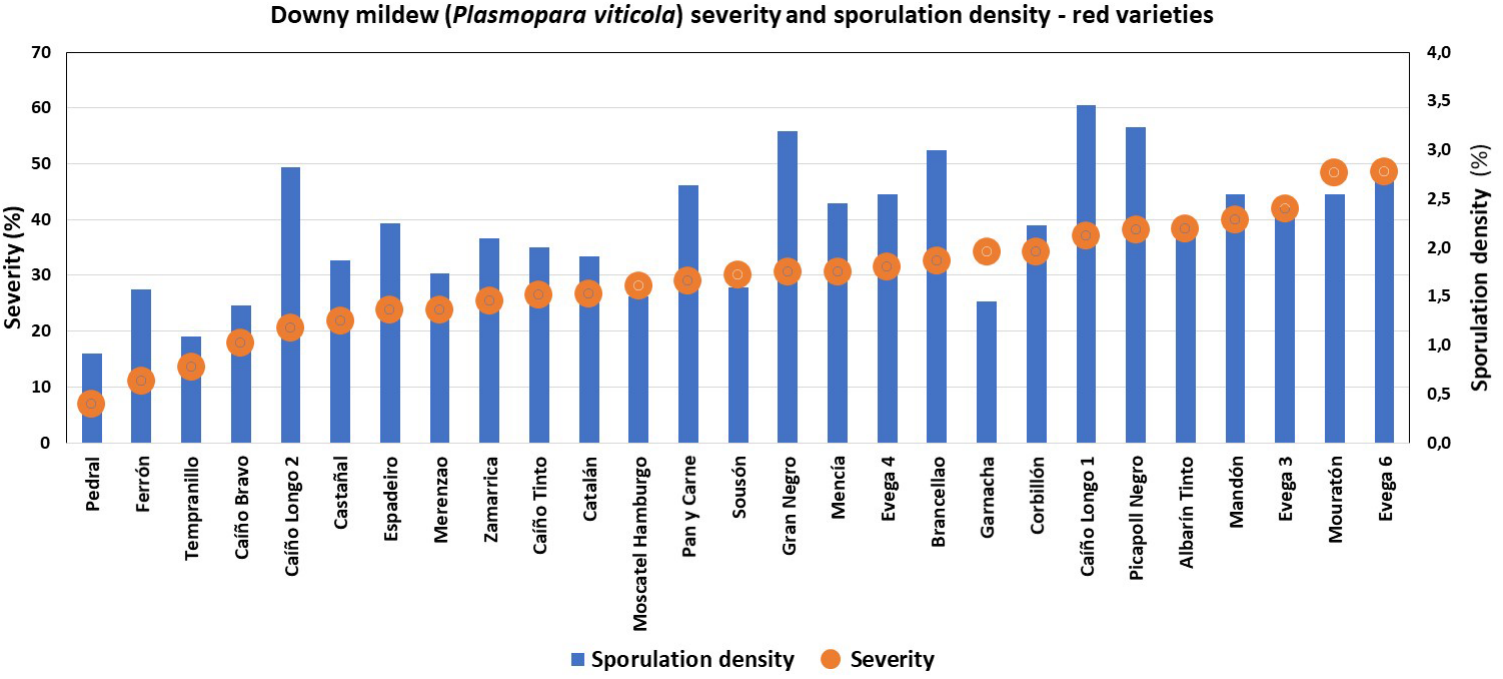
Degree of susceptibility to downy mildew (*Plasmopara viticola*) of different red grape varieties.

Great diversity was found, since only six of the characterized ampelographic and ampelometric parameters were homogeneous for all varieties (OIV 001, OIV 005, OIV 011, OIV 056, OIV 089, and OIV 151). Concerning the susceptibility degree to *P. viticola*, all varieties displayed values of 100% downy mildew incidence, except for “Batoca”, “Caíño Longo 2”, “Catalán”, “Dona Branca”, and “EVEGA 4” with a 95% incidence, and “Albariño” with an 82% incidence (data not shown). Regarding downy mildew severity and sporulation density, the one-way ANOVA showed significant differences among varieties, with “Torrontés”, “EVEGA 6” (synonym “DOCE”), and “Mouratón” showing the highest disease severities while “Ferrón” and “Pedral” showed the lowest ones (**Supplementary Table S2**; **Figures 2**, **3**). Moreover, “Blanca de Monterrei”, “Caíño Longo 1”, and “Albilla do Avia” showed the highest sporulation densities while “Catalán” (a direct producer hybrid) and “Pedral” showed the lowest ones (**Supplementary Table S3**, **Figures 2**, **3**).

### 3.4 Phenological stages and berry quality parameters

**Figure 4:**
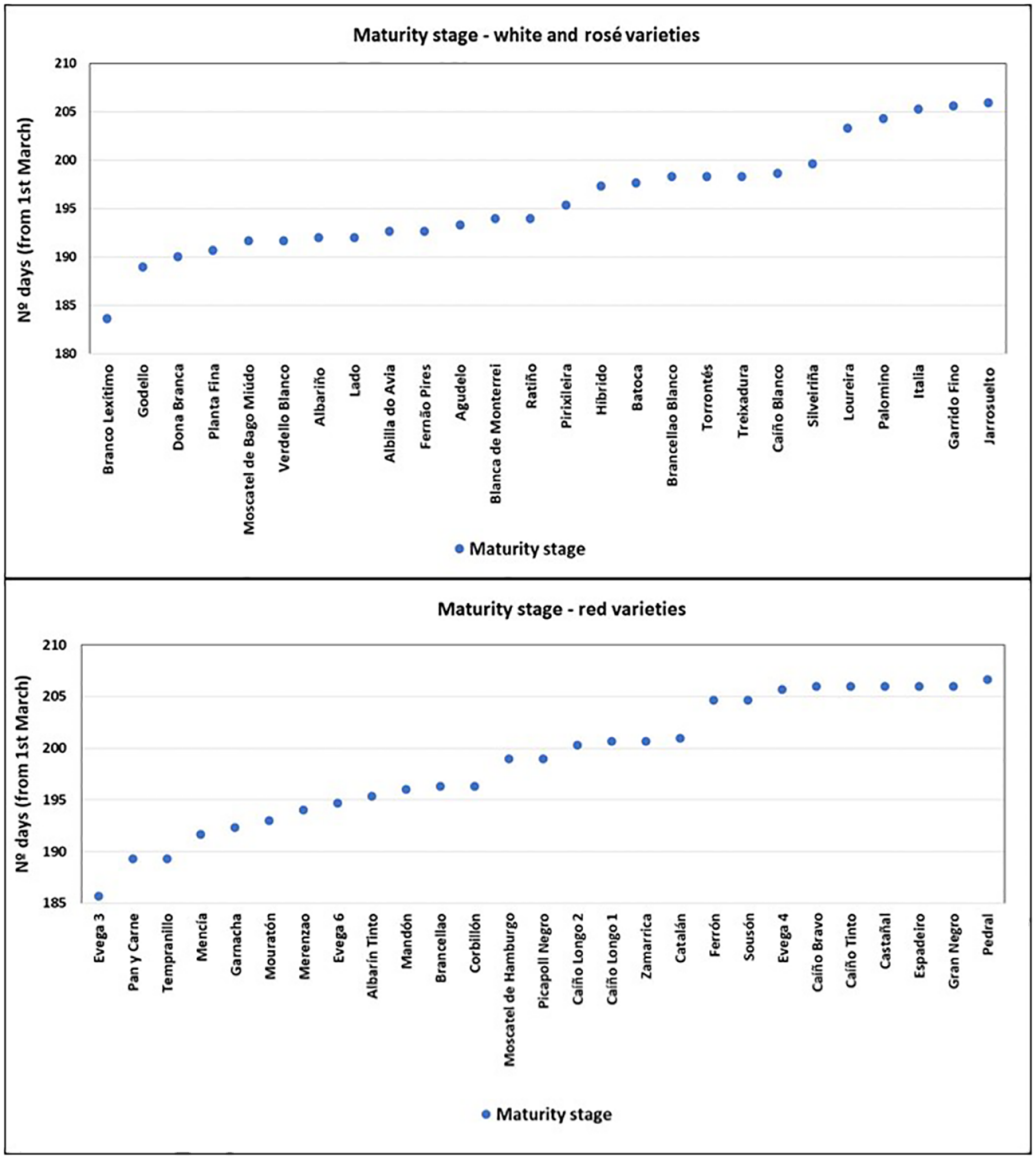
Days to reach the maturity stage (from 1^st^ of March).

A high diversity was found between varieties. In terms of phenology, there was a 23-day difference between the earliest and the latest ripening variety, considering the three vintages’ average values (**Figure 4**).

No significant differences were found between varieties in terms of GDDs required for budburst (**Supplementary Table S4**). At flowering, ‘Catalán’ required less GDD and ‘Sousón’ required more GDDs than the rest of the varieties (**Supplementary Table S5**). At veraison, there were no significant differences between varieties, except for “Pirixileira” (synonym “Chasselas Cioutat”), “Mouratón”, “Mencía”, and “Merenzao” with low GDDs, and “Caíño Blanco”, “Agudelo”, “Caíño Bravo”, “Ferrón”, “Corbillón”, “Ratiño”, and “Treixadura” with the highest values (**Supplementary Table S6**). “Branco Lexítimo” ripened significantly earlier and “Pedral” showed significantly later ripening (**Supplementary Table S7**). “Branco Lexítimo” and “EVEGA 3” required the lowest GDD values to complete the period from budburst to harvest while “Gran Negro” and “Italia” required the highest ones (**Supplementary Table S8**).

Great diversity was also found in berry composition, with differences of 6.37 g L^−1^ (tartaric acid) between the most and the least acid variety (“Ferrón and “Pirixileira”, respectively).

Differences of 6°Brix between varieties were also found (“Blanca de Monterrei” and “Verdello Blanco”). The ANOVA performed with the berry data showed significant differences for all parameters except for the pH (**Supplementary Tables S9**-**S13**). Concerning total acidity, Galician varieties, “Branco Lexítimo”, “Caíño Longo 1”, “Caíño Longo 2”, “Caíño Tinto”, “Ferrón”, “Loureira”, “Ratiño”, and “Zamarrica”, together with “Catalán”, showed the highest values, whereas two foreign varieties, “Palomino” and “Pirixileira”, showed the lowest ones. Regarding the tartaric acid, the highest values were recorded for “Branco Lexítimo”, “Loureira”, and “Zamarrica”, while the lowest values were found for “Caíño Bravo”, “Castañal”, “Espadeiro”, and “Sousón”, all of them of Galician origin. “EVEGA 3”, “Merenzao”, “Pan y carne”, and “Verdello Blanco” achieved the highest °Brix, while “Blanca de Monterrei” and “Brancellao Blanco” achieved the lowest values (**Supplementary Tables S9**-**S13**).

### 3.5 Genetic and phenotypic variation

A PCA based on the 57 ampelographic and 17 ampelometric parameters data studied, together with the qualitative data obtained through the leaf disc test (disease incidence, sporulation density, and disease severity to *P. viticola*), was performed over 53 genotypes. The first axis of the PCA accounted for 7.55% of the total variation, mainly due to the OIV 602 (length of vein N2), the OIV 617 (length between the tooth tip of N2 and the tooth tip of the first secondary vein of N2 in mature leaf), the OIV 615 (width of tooth of N4 in mature leaf), and other ampelometric parameters.

The second axis explained 7.25% of the total variation, based mainly on the berry width and length, and the area and intensity of the anthocyanin coloration on bud scales (OIV parameters 221, 220, 015-1, and 015-2). The third axis accounted for 6.07% of the variation, with this axis being mainly associated with the length of the primary bunch peduncle, the length of vein N4 in mature leaf, and the length and the berry skin color (OIV parameters 206, 604, 225, and 220).

**Figure 5:**
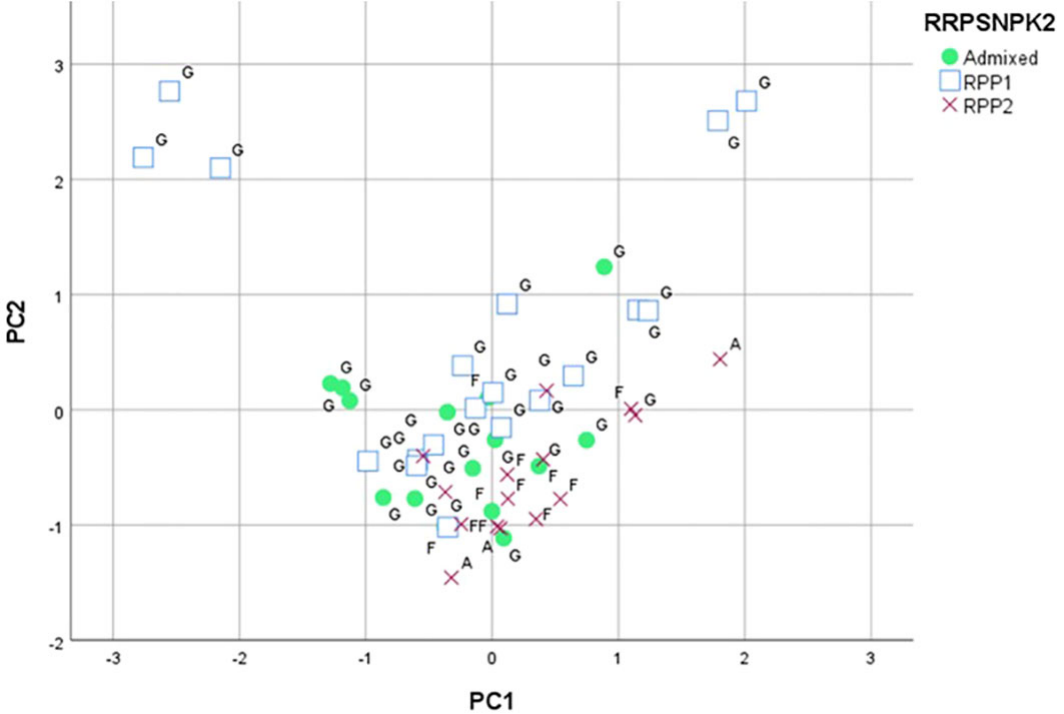
Principal component analysis based on the ampelographic, ampelometrical and leaf disc test data classified for K=2 (RPP1 and RPP2) by SNPs and SSRs (Díaz-Losada et al., 2012). Letters indicate RPPs based on SSR data (G: RPP1; F: RPP2; A: Admixed). The markers (square, circle, cross) indicate RPPs based on SNP data.

The parameters measured for the assessment of susceptibility to *P. viticola* did not have a significant contribution to the variance explained on these three axes. The projection on a plane of the first two axes (**Figure 5**) allowed grouping the varieties included in RPP2 and most of the individuals of the admixed population (Díaz-Losada et al., 2012), which were placed in the negative side of the second axis, also confirmed with the set of SNPs. The negative side of the first axis and the positive side of the second axis grouped the Galician varieties “Albariño”, “Caíño Blanco”, “Lado”, “Ratiño”, and “Verdello Blanco”; adding the following varieties if only the negative side of the first axis is considered: “Agudelo”, “Albarín Tinto”, “Branco Lexítimo”, “Corbillón”, “Espadeiro”, “EVEGA 3”, “EVEGA 4”, “Fernão Pires”, “Godello”, “Loureira”, “Moscatel de Bago Miúdo”, “Picapoll Negro”, and “Silveiriña” (**Supplementary Table S14**).

**Figure 6:**
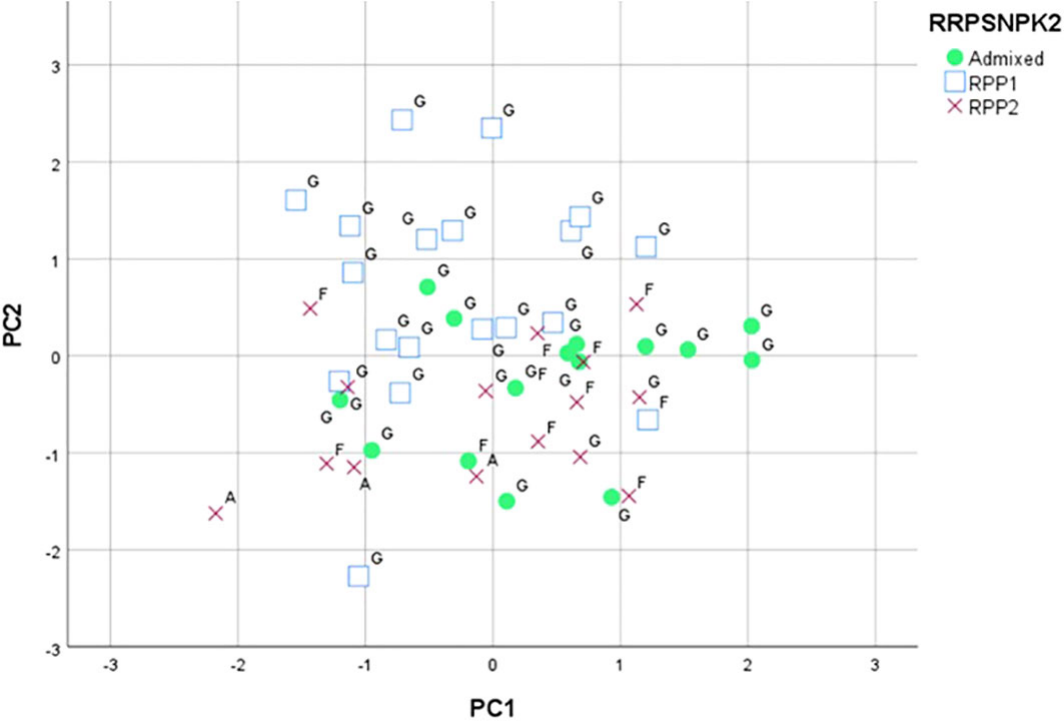
Principal component analysis based phenological and berry quality data classified for K=2 (RPP1 and RPP2) by SNPs and SSRs (Díaz-Losada et al., 2012). Letters indicate RPPs based on SSR data (G: RPP1; F: RPP2; A: Admixed). The markers (square, circle, cross) indicate RPPs based on SNP data.

The PCA based on phenology and berry quality parameters carried out on the data of 53 genotypes made it possible to discriminate the groups represented by the different genetic populations. The first axis of the graphic obtained accounted for 31.3% of the total variation, mainly due to the °Brix, the maturation date, and the duration of the vegetative cycle. The second axis explained 27.8% of the total variation, mainly due to the total acidity and the veraison date. The third axis accounted for 13.0% of the variation, being mainly associated with the tartaric acid and the budburst and flowering dates. The projection of the two first axes (**Figure 6**) separated most of the RPP1 population varieties (Díaz-Losada et al., 2012) in the positive side of the second axis. This side grouped Galician varieties classified in the RPP1 by both SNPs and SSRs: “Albariño”, “Caíño Blanco”, “Caíño Bravo”, “Caíño Longo 1”, “Caíño Longo 2”, “Caíño Tinto”, “Ferrón”, “Loureira”, “Ratiño”, and “Zamarrica” (**Supplementary Table S14**). Again, two Galician varieties classified in the RPP1 by SSRs (Díaz-Losada et al., 2012) showed the highest positive values for the first axis, “EVEGA 3” and “Verdello Blanco”. On the other side, the RPP2 population varieties were mainly in the negative side of the second axis and the admixed varieties were mainly in the positive side of the first axis.

## 4 Discussion

The conservation of grapevine varietal diversity in germplasm banks prevents their disappearance and allows them to be used as a future source of genes to face new pathogens or climatic constraints, as well as provide a possibility to diversify the wine market (Gisbert et al., 2018; Pérez-Navarro et al., 2019). In this sense, many genetic resources at risk of extinction are being prospected, collected, and evaluated in many traditional wine-producing countries (García-Muñoz et al., 2014; Mena et al., 2014; Sancho-Galán et al., 2020), and the European community, aware of their importance, has funded several projects in recent years (GenRes081, GrapeGen06, and GrapeNet), aimed at their conservation and characterization.

In the present study, ANOVA, principal components, and genetic marker analyses were used to identify and classify the varietal diversity of the EVEGA Grapevine Germplasm Bank. Ampelographic descriptions, together with the identification of plant material using DNA markers, are the first steps in the characterization of grapevine germplasm collections (Muganu et al., 2009; Rakonjac et al., 2014; Zombardo et al., 2021). High diversity was found at a morphological level, as only 6 out of 74 ampelographic and ampelometric parameters studied were homogeneous for all varieties. This high morphological diversity has also been reported in other collections (Lamine et al., 2014; Zinelabidine et al., 2014; Khalil et al., 2017; Abiri et al., 2020; Milišić et al., 2021). The variation explained by the first three axes of the PCA using ampelographic, ampelometric, and leaf disc test data was low and similar to that obtained by Merkouropoulos et al. (2015) in an ampelographic study in a Greek grapevine collection. The projection of the first two axes on a plane allowed grouping the varieties included in the RPP2 and in the admixed population established in a previous work carried out with 21 microsatellite markers in the EVEGA Collection (Díaz-Losada et al., 2012). The inclusion of *P. viticola* susceptibility data in the PCA did not have a significant effect on the contribution to diversity on the first axis compared to ampelographic and ampelometric parameters. Nevertheless, the ANOVA performed on the *P. viticola* susceptibility data showed significant differences between varieties (Boso and Kassemeyer, 2008; Jürges et al., 2009; Boso et al., 2014; Gaforio et al., 2015; Bove and Rossi, 2020). This highlights the importance of grapevine collections as valuable resources for large-scale germplasm screening to identify varieties less susceptible to fungal diseases, as seen in the case of powdery mildew (Gaforio et al., 2011).

European Union viticulture employs 68,000 tons of fungicides annually for phytosanitary control. It is estimated that only 0.1% of these fungicides reach the pathogen, with the rest contributing to environmental contamination (Buonassisi et al., 2017). Therefore, having a large reservoir of grapevine genetic resources and assessing their tolerance to fungal diseases is important to select the most suitable varieties to plant, according to the climatic conditions of a specific geographical region, in order to reduce the fungicides applied. A 5%–20% increase in downy mildew disease pressure has been predicted across Europe by 2030, with the exception of some areas in Spain, Germany, France, and Italy, where the pressure will remain stable. A small increase is also expected in Northern Spain in 2050 (Bregaglio et al., 2013). This is important to consider in the Galician region, which is characterized by mild temperatures and high rainfall, leading to a high incidence of fungal diseases.

All evaluated varieties of the EVEGA Collection were considered susceptible to downy mildew, with an incidence of more than 80%. As previously shown, *Vitis vinifera* L. varieties are highly susceptible to *P. viticola*, although there are different susceptibility degrees between varieties and even between clones (Boso and Kassemeyer, 2008; Van Leeuwen et al., 2013; Gaforio et al., 2015). Only one exception to this susceptibility has recently been found in *Vitis vinifera* L., shown by the Georgian variety “Mgaloblishvili”, which limits fungal growth and sporulation through the synthesis of antimicrobial compounds and the deposition of structural barriers (Toffolatti et al., 2018). Gaforio et al. (2015) evaluated 158 varieties from the National Germplasm Bank of “El Encín” (Spain), in the field and with leaf disc test, some of the varieties that are also included in our study. They found a good correlation between susceptibility results in the field and with leaf disc test. With the latter methodology, they detected six varieties of *Vitis vinifera* L. with a very high level of resistance (low incidence of disease), three of them from Galicia (“Caíño Tinto”, “Loureira”, and “Sousón”). All the varieties tested in our study showed very high susceptibility, except “Albariño”, with low susceptibility. Boso et al. (2014) found a 100% incidence of downy mildew in a study with 13 varieties grown in Galicia, 9 of them included in this study. Results obtained in this study were more in line with them than with those of Gaforio et al. (2015). A high sporulation density in “Albariño” was obtained, as it was also recorded by Boso et al. (2014).

In terms of phenology and berry quality, a large variation was also found in parameters such as budburst and ripening dates, total acidity, °Brix, pH, and organic acids. Differences have also been found in other collections, such as that of “El Encín” (Spain), where 18 varieties from the Balearic Islands were agronomic and oenologically evaluated, confirming the importance of minor varieties in the development of new wines (García-Muñoz et al., 2014). In the same collection, large differences were recorded in a long-term study on 43 varieties (Muñoz-Organero et al., 2022). In the Russian ampelographic collection of “Novocherkassk”, a study carried out with eight Georgian and three Dagestan varieties found differences in phenology, sugar content, and titratable acidity (Ganich and Naumova, 2020). Large diversity in soluble solids, anthocyanins, and phenolic content was noted in a study performed on 91 Greek accessions from the “AUTh’s” ampelographic collection (Merkouropoulos et al., 2015). Khalil et al. (2017) also observed high variation in both total soluble solids and titratable acidity traits in a Syrian grapevine collection. Referring to the budburst dates, and contrary to what happened with the other phenological variables studied, the amplitude shown between the data from different varieties was not reflected in the significant differences among varieties in the statistical analyses, which may be due to the fact that, despite the ease of using the GDD, it may not be the most suitable method for budburst estimation because it does not take into account factors such as the dormancy period or the use of daily temperature accumulation, with a base temperature (T0) of 10°C below at which the plant is not considered to be active, with this last factor being the one that seems to be the greatest estimation error. Alternative models such as the BRIN model with a T0 of 5°C and the post-dormancy period estimation using sum of hourly temperatures (growing degree hours—GDH) instead of daily temperatures, or a GDD model with T0 of 5°C instead of 10°C, seem to be much more accurate for the estimation of budburst dates (García de Cortázar-Atauri et al., 2009).

The SNPs confirmed the subgroup of varieties from Western Galicia previously defined with SSRs, RPP1a (Díaz-Losada et al., 2012), also grouping those varieties in the same cluster, in which “Caiño Bravo” could be the key variety as also defined by Augusto et al. (2021) in Northern Portugal. However, differences were found between the population structures defined by SNPs and SSRs, which could be a consequence of the properties of the molecular marker itself. This showed that further analysis and interpretation are necessary (Emanuelli et al., 2013; Cunha et al., 2016; Cunha et al., 2020). It is noteworthy that only few studies have delved into the correlation between molecular and ampelographic or biochemical data, and in general, no correlation has been found (Knezović et al., 2017; Labagnara et al., 2018; Chehade et al., 2022). Although Muccillo et al. (2014) established a high correlation between SSR and several phenylpropanoid molecules, the study was only performed on seven varieties not established in a germplasm collection. In the case of this study, PCAs performed with ampelographic, ampelometric, leaf disc test, phenology, and berry quality data allowed grouping some of the same varieties included in genetic populations established through SNPs and in a previous study by Díaz-Losada et al. (2012) based on SSR data.

The exhaustive study of the varieties included in the EVEGA Germplasm Bank has confirmed the wide existing variability. This will allow a possible future use of the different varieties in breeding programs to adapt to climate change, to reduce the application of phytosanitary products, or to obtain new wines.

## Data availability statement

The original contributions presented in the study are included in the article/**Supplementary Material**, further inquiries can be directed to the corresponding authors.

## Author contributions

ÁD-F: Data curation, Methodology, Writing – original draft, Writing – review & editing. ML: Formal Analysis, Writing – review & editing, Writing – original draft. SP-L: Formal Analysis, Methodology, Writing – original draft, Writing – review & editing. JI: Data curation, Writing – review & editing. ED-L: Conceptualization, Data curation, Funding acquisition, Methodology, Project administration, Writing – review & editing.
