# Supplementary material for: Screening of Galician grapevine varieties by SNPs, phenotypic traits, and phytopathology
Source: Front Plant Sci. 2024 Feb 16;15:1359506. doi: 10.3389/fpls.2024.1359506 (PMC10904527; doi:10.3389/fpls.2024.1359506)
Supplement: Supplementary file 1 [file DataSheet_1.docx]

**Table S1**: SNP data for the 27 varieties

| **SNP** | ‘Albilla do Avia ’ | 'Blanca de Galicia' | ‘Blanca de Monterrei’ | 'Blanca de Ribeiras' | ‘Caíño Longo 1’ | ‘Caíño Longo 2 ’ | ‘Catalán’ | 'Cruce Paco' | ‘Dona Branca’ | ‘EVEGA 3’ | ‘EVEGA 4’ | ‘EVEGA 5’ | ‘EVEGA 6’ |
| --- | --- | --- | --- | --- | --- | --- | --- | --- | --- | --- | --- | --- | --- |
| **SNP1003_336** | CC | CC | CC | AA | AC | CC | AC | AA | CC | CC | CC | CC | AC |
| **SNP1015_67** | AG | GG | AG | GG | GG | GG | GG | GG | AG | GG | GG | AG | GG |
| **SNP1027_69** | CT | CT | CC | CC | CC | CC | TT | CC | CC | CT | CC | CT | CC |
| **SNP1035_226** | CT | CT | CC | CT | TT | CT | TT | TT | TT | TT | TT | CT | CC |
| **SNP1079_58** | GG | AA | AG | GG | GG | AG | GG | AG | AG | AG | GG | AG | AA |
| **SNP1119_176** | CC | AC | AC | -- | AA | AA | AA | CC | AC | AA | AC | AA | AC |
| **SNP1127_70** | GT | GG | GT | GT | GT | GT | GG | GT | GG | GT | GT | GG | GT |
| **SNP1157_64** | AT | TT | TT | TT | AA | AT | AT | TT | TT | TT | TT | TT | TT |
| **SNP1215_138** | CT | CT | CT | TT | CT | CT | TT | TT | CT | CT | TT | CT | CT |
| **SNP1229_219** | CC | CC | CC | CC | CG | CG | CG | CC | CC | CG | CG | CC | CG |
| **SNP1323_155** | CC | AC | CC | AC | CC | CC | AC | AC | CC | AC | AA | CC | AA |
| **SNP1347_100** | AG | AG | AG | GG | AG | AG | GG | GG | AG | AG | AG | AG | -- |
| **SNP1349_174** | AG | AG | GG | AG | GG | GG | AG | AG | AG | AG | GG | AG | GG |
| **SNP1399_81** | AA | AA | AA | AA | AA | AG | AA | AG | AA | AG | AA | AA | AA |
| **SNP1411_565** | AA | AT | TT | -- | AT | TT | TT | AT | TT | TT | AT | TT | TT |
| **SNP1445_218** | AG | AG | AG | AA | AG | GG | GG | AG | AA | AG | AA | GG | AA |
| **SNP1453_40** | GG | AA | AG | AA | AA | AG | AG | AA | AG | AA | AA | AA | AG |
| **SNP1471_179** | TT | CT | TT | -- | -- | -- | CT | -- | TT | -- | -- | CT | CT |
| **SNP1513_153** | CT | CC | CT | CT | CT | CT | CC | TT | TT | CC | CT | TT | CT |
| **SNP191_100** | CC | CT | CC | CC | CC | CC | CT | CT | CC | CT | CT | CC | -- |
| **SNP197_82** | CC | AC | CC | -- | AC | AC | AC | AA | AC | AA | AC | CC | CC |
| **SNP227_191** | AC | CC | AA | -- | -- | -- | AA | -- | AC | -- | -- | AA | AC |
| **SNP259_199** | AT | AT | TT | AT | TT | TT | TT | AT | AT | AT | TT | TT | TT |
| **SNP269_308** | GG | AG | GG | -- | GG | AG | AG | AA | AG | GG | GG | AG | AG |
| **SNP325_65** | AA | AA | AA | AA | AT | AT | AA | AT | AA | AT | AT | AA | AT |
| **SNP425_205** | AA | AA | AA | AA | AA | AA | AA | AC | AA | AA | AA | AA | AA |
| **SNP447_244** | TT | CC | CT | CC | CT | CT | CC | CT | CC | CT | CC | CC | CT |
| **SNP555_132** | AC | CC | AA | CC | AC | CC | CC | AC | AC | CC | AA | AA | -- |
| **SNP579_187** | TT | TT | CT | -- | TT | TT | TT | TT | CT | CT | CT | CT | CT |
| **SNP581_114** | AG | AG | AG | GG | GG | GG | AA | AG | AG | AG | AG | GG | -- |
| **SNP593_149** | TT | TT | TT | CT | CT | TT | CC | CC | TT | TT | CC | TT | TT |
| **SNP613_315** | CT | CT | TT | CC | CT | CT | CC | CC | CT | CT | CC | CT | CC |
| **SNP697_296** | AA | AA | AG | AA | AA | AA | AA | AA | AG | AA | AA | AA | AA |
| **SNP819_210** | TT | TT | AT | AT | AT | AT | TT | AT | AT | TT | AT | TT | AA |
| **SNP829_281** | AG | GG | AG | AG | AG | AA | GG | AG | AG | GG | AG | GG | AA |
| **SNP873_244** | TT | CC | CT | TT | TT | CT | TT | -- | CC | CT | CT | CT | -- |
| **SNP879_308** | AG | AG | AA | -- | AA | AG | AA | AA | AG | AG | AA | AA | AA |
| **SNP895_382** | AT | AT | AT | AT | TT | AT | AT | AA | TT | TT | TT | AT | TT |
| **SNP945_88** | AA | AG | AG | -- | AA | AA | AG | AA | AG | AG | AA | AG | AG |
| **SNP947_288** | GG | AG | GG | AG | GG | GG | AG | AG | GG | AG | AG | GG | AA |
| **Vvi_10113** | AA | AA | AG | GG | AA | AA | AG | AG | AA | AA | AA | AA | AA |
| **Vvi_10329** | TT | TT | CT | -- | -- | -- | TT | -- | CT | -- | -- | CC | TT |
| **Vvi_10353** | GG | GG | AG | GG | AA | AG | GG | AG | GG | GG | GG | GG | AG |
| **Vvi_10992** | AT | AT | AT | AA | AA | AA | AA | AA | AT | AA | AT | AT | AT |
| **Vvi_12882** | TT | CT | TT | TT | TT | CT | CT | CC | TT | TT | CT | TT | TT |
| **Vvi_1617** | CC | AC | AC | AC | CC | CC | CC | AC | CC | AC | CC | AA | AC |
| **Vvi_9227** | TT | AT | AT | TT | TT | TT | TT | TT | AT | TT | AT | TT | AT |
| **Vvi_9920** | GG | GG | GG | GG | AG | AG | GG | GG | GG | GG | GG | GG | GG |

**Table S1 (Continuation)**: SNP data for the 27 varieties

| **SNP** | **'Malvasía Bianca'** | **'Moscatel Rubio'** | **'Mosteiro 14'** | **'Negrón de Aldán 1 '** | **'Olho de Pargo'** | **'Ollo de Sapo'** | **‘Pedral’** | **‘Ratiño’** | **'Tinta de Bares'** | **‘Treixadura José Hermo’** | **‘Verdello Blanco’** | **'Verdello Sebio'** | **'Xafardán’** | **‘Zamarrica ’** |
| --- | --- | --- | --- | --- | --- | --- | --- | --- | --- | --- | --- | --- | --- | --- |
| **SNP1003_336** | CC | CC | CC | CC | AC | AC | AC | AC | CC | AC | CC | AC | AC | AC |
| **SNP1015_67** | GG | GG | GG | AG | AG | GG | GG | GG | GG | GG | GG | AG | GG | GG |
| **SNP1027_69** | CC | CC | CT | CC | CT | CT | CT | CT | CC | CC | CC | CT | TT | CC |
| **SNP1035_226** | CT | TT | TT | CT | TT | TT | TT | TT | TT | CT | CT | TT | CT | CT |
| **SNP1079_58** | AG | AA | AG | AG | AG | GG | AG | GG | GG | GG | GG | AG | AG | AG |
| **SNP1119_176** | AC | AC | CC | CC | AA | AA | AC | AA | AC | AA | AC | AC | AC | AA |
| **SNP1127_70** | GT | GT | GT | GT | TT | TT | GG | TT | GT | GT | TT | GT | GT | TT |
| **SNP1157_64** | TT | AT | TT | AT | TT | TT | TT | TT | TT | AT | TT | AT | TT | TT |
| **SNP1215_138** | CT | CT | CT | CT | CC | TT | TT | TT | TT | TT | CT | CT | TT | TT |
| **SNP1229_219** | CC | CG | CC | CG | CG | CC | CG | CC | CG | -- | CC | CC | CG | CG |
| **SNP1323_155** | CC | AC | AC | AC | AC | CC | AC | CC | AA | AC | CC | AC | AC | AC |
| **SNP1347_100** | AG | AG | AG | AG | AG | AG | AG | AG | AG | GG | AG | AG | AG | AG |
| **SNP1349_174** | AG | AG | GG | AG | GG | AG | GG | AG | AG | GG | AA | AG | GG | AG |
| **SNP1399_81** | AA | AA | AG | AA | AA | AA | AG | AA | AA | AA | AG | AG | AA | AA |
| **SNP1411_565** | TT | AT | AT | TT | AA | AT | AT | AT | TT | TT | TT | AT | TT | TT |
| **SNP1445_218** | AA | GG | AG | AG | AG | AG | GG | AG | AG | AG | AG | AA | AG | AG |
| **SNP1453_40** | AG | AG | AA | AG | AG | AA | AG | AA | AG | AA | GG | AG | AA | AA |
| **SNP1471_179** | CT | TT | CT | TT | TT | TT | TT | TT | -- | -- | -- | TT | CT | TT |
| **SNP1513_153** | CT | CT | CC | TT | TT | CT | TT | CT | CT | TT | CT | CC | CC | CT |
| **SNP191_100** | CC | CC | CC | CC | CC | CT | CC | CT | CT | CT | CC | CC | CT | CT |
| **SNP197_82** | AC | AC | AC | CC | CC | AC | AA | AC | AA | AC | AC | AC | AC | AC |
| **SNP227_191** | AC | AA | AA | AA | AA | AC | CC | AC | -- | -- | -- | AA | AC | AA |
| **SNP259_199** | TT | AT | TT | AT | AT | TT | AT | TT | AT | TT | AT | AA | AT | AT |
| **SNP269_308** | AA | AG | GG | AG | AG | GG | AG | GG | GG | AG | GG | GG | GG | AG |
| **SNP325_65** | AA | AA | AT | AA | AT | AA | AA | AA | AT | AA | AA | AT | AA | AA |
| **SNP425_205** | AA | AA | AA | AA | AA | AA | AA | AA | AA | AA | AA | AA | AA | AA |
| **SNP447_244** | CC | CC | CT | CC | CT | CC | CT | CC | TT | CC | CT | CC | CC | CT |
| **SNP555_132** | CC | AC | AA | AC | AA | AC | AC | AC | AC | AA | AC | AA | AC | AC |
| **SNP579_187** | TT | TT | TT | CT | CT | TT | TT | TT | TT | TT | TT | TT | CT | TT |
| **SNP581_114** | AG | AG | AG | AG | AG | AG | AG | AG | GG | AG | GG | GG | AG | GG |
| **SNP593_149** | CC | CT | CT | CT | TT | TT | TT | TT | CT | CC | TT | TT | TT | CT |
| **SNP613_315** | CC | CT | CT | CT | CC | CT | CT | CT | CT | CT | CT | CC | CC | CT |
| **SNP697_296** | AA | AA | AA | AG | AG | AA | AG | AA | AA | AA | AA | AA | AA | AG |
| **SNP819_210** | AT | AT | TT | AA | AT | AT | AT | AT | TT | TT | AT | AA | TT | TT |
| **SNP829_281** | AG | GG | AG | GG | AG | AG | GG | AG | GG | AA | GG | GG | AG | AG |
| **SNP873_244** | CT | CT | CC | CC | CT | CT | CC | CT | CT | CT | CC | TT | CC | CT |
| **SNP879_308** | AA | AG | AA | AA | AA | AA | AG | AA | AG | AA | AG | AA | AG | AG |
| **SNP895_382** | AT | AT | AT | AT | AA | TT | TT | TT | TT | TT | TT | AA | TT | AA |
| **SNP945_88** | GG | AG | AG | AG | AG | AG | AA | AG | AG | AA | AA | AG | AG | AG |
| **SNP947_288** | AG | AA | GG | GG | GG | AA | GG | AA | AG | GG | GG | AG | AG | AG |
| **Vvi_10113** | AA | AG | AA | GG | AA | AG | AA | AG | AG | AA | AG | AG | AG | AG |
| **Vvi_10329** | TT | CT | TT | CT | -- | TT | CT | TT | -- | -- | -- | CT | TT | -- |
| **Vvi_10353** | GG | GG | GG | GG | GG | AG | AG | AG | GG | GG | GG | GG | GG | AG |
| **Vvi_10992** | AA | AT | AA | TT | AT | AA | AA | AA | AA | AA | AA | AA | AT | AA |
| **Vvi_12882** | TT | CT | CT | CT | CT | CT | CT | CT | TT | CT | CT | TT | TT | TT |
| **Vvi_1617** | CC | AC | CC | AC | CC | AC | AC | AC | AC | AC | CC | AC | AA | CC |
| **Vvi_9227** | AT | TT | AT | AT | AT | AT | AT | AT | TT | AT | AT | AT | AT | TT |
| **Vvi_9920** | GG | GG | GG | GG | GG | GG | GG | GG | AG | GG | GG | GG | AG | AG |

**Table S2**: Mean values and statistical differences in mildew severity (sporulation area) between varieties (p < 0.05).

| **Variety** | **Average** | **Groups** | | | | | | | | | | | | | | | | | | | | | | |  |  |
| --- | --- | --- | --- | --- | --- | --- | --- | --- | --- | --- | --- | --- | --- | --- | --- | --- | --- | --- | --- | --- | --- | --- | --- | --- | --- | --- |
| ‘Torrontés’ | 61.09 | a |  |  |  |  |  |  |  |  |  |  |  |  |  |  |  |  |  |  |  |  |  |  | |  |
| ‘EVEGA 6’ | 48.53 |  | b |  |  |  |  |  |  |  |  |  |  |  |  |  |  |  |  |  |  |  |  |  | |  |
| ‘Mouratón’ | 48.40 |  | b |  |  |  |  |  |  |  |  |  |  |  |  |  |  |  |  |  |  |  |  |  | |  |
| ‘Treixadura’ | 45.29 |  | b | c |  |  |  |  |  |  |  |  |  |  |  |  |  |  |  |  |  |  |  |  | |  |
| ‘Moscatel de Bago Miúdo’ | 43.93 |  | b | c | d |  |  |  |  |  |  |  |  |  |  |  |  |  |  |  |  |  |  |  | |  |
| ‘Blanca de Monterrei’ | 43.86 |  | b | c | d |  |  |  |  |  |  |  |  |  |  |  |  |  |  |  |  |  |  |  | |  |
| ‘Fernao Pires’ | 42.51 |  | b | c | d | e |  |  |  |  |  |  |  |  |  |  |  |  |  |  |  |  |  |  | |  |
| ‘EVEGA 3’ | 41.99 |  | b | c | d | e | f |  |  |  |  |  |  |  |  |  |  |  |  |  |  |  |  |  | |  |
| ‘Mandón’ | 40.03 |  | b | c | d | e | f | g |  |  |  |  |  |  |  |  |  |  |  |  |  |  |  |  | |  |
| ‘Albarín Tinto’ | 38.36 |  | b | c | d | e | f | g | h |  |  |  |  |  |  |  |  |  |  |  |  |  |  |  | |  |
| ‘Picapoll Negro’ | 38.14 |  | b | c | d | e | f | g | h | i |  |  |  |  |  |  |  |  |  |  |  |  |  |  | |  |
| ‘Verdello Blanco’ | 38.05 |  | b | c | d | e | f | g | h | i |  |  |  |  |  |  |  |  |  |  |  |  |  |  | |  |
| ‘Caíño Longo 1’ | 37.05 |  | b | c | d | e | f | g | h | i | j |  |  |  |  |  |  |  |  |  |  |  |  |  | |  |
| ‘Planta Fina’ | 36.28 |  |  | c | d | e | f | g | h | i | j | k |  |  |  |  |  |  |  |  |  |  |  |  | |  |
| ‘Garrido Fino’ | 35.21 |  |  | c | d | e | f | g | h | i | j | k | l |  |  |  |  |  |  |  |  |  |  |  | |  |
| ‘Corbillón’ | 34.22 |  |  | c | d | e | f | g | h | i | j | k | l | m |  |  |  |  |  |  |  |  |  |  | |  |
| ‘Albilla do Avia’ | 34.16 |  |  | c | d | e | f | g | h | i | j | k | l | m |  |  |  |  |  |  |  |  |  |  | |  |
| ‘Garnacha’ | 34.14 |  |  | c | d | e | f | g | h | i | j | k | l | m | n |  |  |  |  |  |  |  |  |  | |  |
| ‘Lado’ | 33.66 |  |  |  | d | e | f | g | h | i | j | k | l | m | n |  |  |  |  |  |  |  |  |  | |  |
| ‘Brancellao’ | 32.62 |  |  |  | d | e | f | g | h | i | j | k | l | m | n | o |  |  |  |  |  |  |  |  | |  |
| ‘Batoca’ | 31.64 |  |  |  |  | e | f | g | h | i | j | k | l | m | n | o | p |  |  |  |  |  |  |  | |  |
| ‘EVEGA 4’ | 31.53 |  |  |  |  | e | f | g | h | i | j | k | l | m | n | o | p | q |  |  |  |  |  |  | |  |
| ‘Palomino’ | 31.19 |  |  |  |  | e | f | g | h | i | j | k | l | m | n | o | p | q | r |  |  |  |  |  | |  |
| ‘Godello’ | 31.04 |  |  |  |  | e | f | g | h | i | j | k | l | m | n | o | p | q | r |  |  |  |  |  | |  |
| ‘Mencía’ | 30.56 |  |  |  |  |  | f | g | h | i | j | k | l | m | n | o | p | q | r |  |  |  |  |  | |  |
| ‘Gran Negro’ | 30.54 |  |  |  |  |  | f | g | h | i | j | k | l | m | n | o | p | q | r |  |  |  |  |  | |  |
| ‘Sousón’ | 30.00 |  |  |  |  |  |  | g | h | i | j | k | l | m | n | o | p | q | r | s |  |  |  |  | |  |
| ‘Pan y Carne’ | 28.92 |  |  |  |  |  |  | g | h | i | j | k | l | m | n | o | p | q | r | s | t |  |  |  | |  |
| ‘Pirixileira’ | 28.74 |  |  |  |  |  |  | g | h | i | j | k | l | m | n | o | p | q | r | s | t |  |  |  | |  |
| ‘Agudelo’ | 28.19 |  |  |  |  |  |  |  | h | i | j | k | l | m | n | o | p | q | r | s | t |  |  |  | |  |
| ‘Moscatel de Hamburgo’ | 28.15 |  |  |  |  |  |  |  | h | i | j | k | l | m | n | o | p | q | r | s | t | u |  |  | |  |
| ‘Catalán’ | 26.62 |  |  |  |  |  |  |  |  | i | j | k | l | m | n | o | p | q | r | s | t | u |  |  | |  |
| ‘Caíño Tinto’ | 26.46 |  |  |  |  |  |  |  |  |  | j | k | l | m | n | o | p | q | r | s | t | u |  |  | |  |
| ‘Loureira’ | 25.97 |  |  |  |  |  |  |  |  |  | j | k | l | m | n | o | p | q | r | s | t | u |  |  | |  |
| ‘Zamarrica’ | 25.43 |  |  |  |  |  |  |  |  |  |  | k | l | m | n | o | p | q | r | s | t | u |  |  | |  |
| ‘Silveiriña’ | 24.82 |  |  |  |  |  |  |  |  |  |  | k | l | m | n | o | p | q | r | s | t | u | v |  | |  |
| ‘Dona Branca’ | 24.54 |  |  |  |  |  |  |  |  |  |  |  | l | m | n | o | p | q | r | s | t | u | v |  | |  |
| ‘Merenzao’ | 23.83 |  |  |  |  |  |  |  |  |  |  |  | l | m | n | o | p | q | r | s | t | u | v |  | |  |
| ‘Jarrosuelto’ | 23.76 |  |  |  |  |  |  |  |  |  |  |  | l | m | n | o | p | q | r | s | t | u | v |  | |  |
| ‘Espadeiro’ | 23.71 |  |  |  |  |  |  |  |  |  |  |  | l | m | n | o | p | q | r | s | t | u | v |  | |  |
| ‘Branco Lexítimo’ | 22.87 |  |  |  |  |  |  |  |  |  |  |  |  | m | n | o | p | q | r | s | t | u | v |  | |  |
| ‘Italia’ | 22.59 |  |  |  |  |  |  |  |  |  |  |  |  |  | n | o | p | q | r | s | t | u | v | w | |  |
| ‘Castañal’ | 21.80 |  |  |  |  |  |  |  |  |  |  |  |  |  |  | o | p | q | r | s | t | u | v | w | |  |
| ‘Caíño Longo 2’ | 20.50 |  |  |  |  |  |  |  |  |  |  |  |  |  |  |  | p | q | r | s | t | u | v | w | |  |
| ‘Brancellao Blanco’ | 20.17 |  |  |  |  |  |  |  |  |  |  |  |  |  |  |  | p | q | r | s | t | u | v | w | |  |
| ‘Caíño Blanco’ | 20.06 |  |  |  |  |  |  |  |  |  |  |  |  |  |  |  |  | q | r | s | t | u | v | w | |  |
| ‘Albariño’ | 19.73 |  |  |  |  |  |  |  |  |  |  |  |  |  |  |  |  |  | r | s | t | u | v | w | |  |
| ‘Ratiño’ | 18.72 |  |  |  |  |  |  |  |  |  |  |  |  |  |  |  |  |  |  | s | t | u | v | w | |  |
| ‘Caíño Bravo’ | 17.86 |  |  |  |  |  |  |  |  |  |  |  |  |  |  |  |  |  |  |  | t | u | v | w | | x |
| ‘Híbrido’ | 16.61 |  |  |  |  |  |  |  |  |  |  |  |  |  |  |  |  |  |  |  |  | u | v | w | | x |
| ‘Tempranillo’ | 13.62 |  |  |  |  |  |  |  |  |  |  |  |  |  |  |  |  |  |  |  |  |  | v | w | | x |
| ‘Ferrón’ | 11.03 |  |  |  |  |  |  |  |  |  |  |  |  |  |  |  |  |  |  |  |  |  |  | w | | x |
| ‘Pedral’ | 6.86 |  |  |  |  |  |  |  |  |  |  |  |  |  |  |  |  |  |  |  |  |  |  |  | | x |

**Table S3**: Mean values and statistical differences in sporulation density between varieties (p < 0.05)

| **Variety** | **Average** | **Groups** | | | | | | | | | | | | | | | | |
| --- | --- | --- | --- | --- | --- | --- | --- | --- | --- | --- | --- | --- | --- | --- | --- | --- | --- | --- |
| ‘Blanca de Monterrei’ | 3.500 | a |  |  |  |  |  |  |  |  |  |  |  |  |  |  |  |  |
| ‘Caíño Longo 1’ | 3.455 | a | b |  |  |  |  |  |  |  |  |  |  |  |  |  |  |  |
| ‘Albilla do Avia’ | 3.364 | a | b | c |  |  |  |  |  |  |  |  |  |  |  |  |  |  |
| ‘Picapoll Negro’ | 3.227 | a | b | c | d |  |  |  |  |  |  |  |  |  |  |  |  |  |
| ‘Gran Negro’ | 3.190 | a | b | c | d |  |  |  |  |  |  |  |  |  |  |  |  |  |
| ‘Brancellao’ | 3.000 | a | b | c | d | e |  |  |  |  |  |  |  |  |  |  |  |  |
| ‘Albariño’ | 2.864 | a | b | c | d | e | f |  |  |  |  |  |  |  |  |  |  |  |
| ‘Caíño Longo 2’ | 2.818 | a | b | c | d | e | f | g |  |  |  |  |  |  |  |  |  |  |
| ‘EVEGA 6’ | 2.727 | a | b | c | d | e | f | g | h |  |  |  |  |  |  |  |  |  |
| ‘Garrido Fino’ | 2.682 | a | b | c | d | e | f | g | h |  |  |  |  |  |  |  |  |  |
| ‘Verdello Blanco’ | 2.682 | a | b | c | d | e | f | g | h |  |  |  |  |  |  |  |  |  |
| ‘Lado’ | 2.636 | a | b | c | d | e | f | g | h | i |  |  |  |  |  |  |  |  |
| ‘Pan y Carne’ | 2.636 | a | b | c | d | e | f | g | h | i |  |  |  |  |  |  |  |  |
| ‘EVEGA 4’ | 2.545 |  | b | c | d | e | f | g | h | i |  |  |  |  |  |  |  |  |
| ‘Mandón’ | 2.545 |  | b | c | d | e | f | g | h | i |  |  |  |  |  |  |  |  |
| ‘Mouratón’ | 2.545 |  | b | c | d | e | f | g | h | i |  |  |  |  |  |  |  |  |
| ‘Batoca’ | 2.500 |  |  | c | d | e | f | g | h | i | j |  |  |  |  |  |  |  |
| ‘Dona Branca’ | 2.500 |  |  | c | d | e | f | g | h | i | j |  |  |  |  |  |  |  |
| ‘Moscatel de Bago Miúdo’ | 2.455 |  |  | c | d | e | f | g | h | i | j | k |  |  |  |  |  |  |
| ‘Mencía’ | 2.455 |  |  | c | d | e | f | g | h | i | j | k |  |  |  |  |  |  |
| ‘Agudelo’ | 2.435 |  |  |  | d | e | f | g | h | i | j | k |  |  |  |  |  |  |
| ‘EVEGA 3’ | 2.409 |  |  |  | d | e | f | g | h | i | j | k | l |  |  |  |  |  |
| ‘Treixadura’ | 2.381 |  |  |  | d | e | f | g | h | i | j | k | l |  |  |  |  |  |
| ‘Godello’ | 2.364 |  |  |  | d | e | f | g | h | i | j | k | l | m |  |  |  |  |
| ‘Fernao Pires’ | 2.318 |  |  |  | d | e | f | g | h | i | j | k | l | m | n |  |  |  |
| ‘Albarín Tinto’ | 2.273 |  |  |  |  | e | f | g | h | i | j | k | l | m | n |  |  |  |
| ‘Espadeiro’ | 2.250 |  |  |  |  | e | f | g | h | i | j | k | l | m | n |  |  |  |
| ‘Corbillón’ | 2.227 |  |  |  |  | e | f | g | h | i | j | k | l | m | n |  |  |  |
| ‘Planta Fina’ | 2.227 |  |  |  |  | e | f | g | h | i | j | k | l | m | n |  |  |  |
| ‘Zamarrica’ | 2.091 |  |  |  |  | e | f | g | h | i | j | k | l | m | n |  |  |  |
| ‘Caíño Tinto’ | 2.000 |  |  |  |  |  | f | g | h | i | j | k | l | m | n | o |  |  |
| ‘Silveiriña’ | 2.000 |  |  |  |  |  | f | g | h | i | j | k | l | m | n | o |  |  |
| ‘Palomino’ | 1.955 |  |  |  |  |  | f | g | h | i | j | k | l | m | n | o |  |  |
| ‘Torrontés’ | 1.955 |  |  |  |  |  | f | g | h | i | j | k | l | m | n | o |  |  |
| ‘Catalán’ | 1.909 |  |  |  |  |  |  | g | h | i | j | k | l | m | n | o |  |  |
| ‘Loureira’ | 1.909 |  |  |  |  |  |  | g | h | i | j | k | l | m | n | o |  |  |
| ‘Ratiño’ | 1.905 |  |  |  |  |  |  | g | h | i | j | k | l | m | n | o |  |  |
| ‘Italia’ | 1.893 |  |  |  |  |  |  |  | h | i | j | k | l | m | n | o |  |  |
| ‘Brancellao Blanco’ | 1.864 |  |  |  |  |  |  |  | h | i | j | k | l | m | n | o | p |  |
| ‘Castañal’ | 1.864 |  |  |  |  |  |  |  | h | i | j | k | l | m | n | o | p |  |
| ‘Jarrosuelto’ | 1.864 |  |  |  |  |  |  |  | h | i | j | k | l | m | n | o | p |  |
| ‘Pirixileira’ | 1.864 |  |  |  |  |  |  |  | h | i | j | k | l | m | n | o | p |  |
| ‘Merenzao’ | 1.737 |  |  |  |  |  |  |  |  | i | j | k | l | m | n | o | p | q |
| ‘Caíño Blanco’ | 1.625 |  |  |  |  |  |  |  |  |  | j | k | l | m | n | o | p | q |
| ‘Branco Lexítimo’ | 1.591 |  |  |  |  |  |  |  |  |  | j | k | l | m | n | o | p | q |
| ‘Sousón’ | 1.591 |  |  |  |  |  |  |  |  |  | j | k | l | m | n | o | p | q |
| ‘Ferrón’ | 1.571 |  |  |  |  |  |  |  |  |  |  | k | l | m | n | o | p | q |
| ‘Moscatel de Hamburgo’ | 1.500 |  |  |  |  |  |  |  |  |  |  |  | l | m | n | o | p | q |
| ‘Garnacha’ | 1.450 |  |  |  |  |  |  |  |  |  |  |  |  | m | n | o | p | q |
| ‘Caíño Bravo’ | 1.409 |  |  |  |  |  |  |  |  |  |  |  |  |  | n | o | p | q |
| ‘Tempranillo’ | 1.089 |  |  |  |  |  |  |  |  |  |  |  |  |  |  | o | p | q |
| ‘Híbrido’ | 0.955 |  |  |  |  |  |  |  |  |  |  |  |  |  |  |  | p | q |
| ‘Pedral’ | 0.909 |  |  |  |  |  |  |  |  |  |  |  |  |  |  |  |  | q |

**Table S4:** Mean values and statistical differences in Growing Degrees Days (GDD) at budburst between varieties (p < 0.05).

| **Variety** | **Average (GDD)** | **Group** |
| --- | --- | --- |
| ‘Ratiño’ | 101.15 | a |
| ‘Torrontés’ | 101.15 | a |
| ‘Mandón’ | 99.13 | a |
| ‘Albariño’ | 93.87 | a |
| ‘Treixadura’ | 93.87 | a |
| ‘Garrido Fino’ | 93.50 | a |
| ‘Híbrido’ | 93.50 | a |
| ‘EVEGA 4’ | 91.48 | a |
| ‘Lado’ | 91.25 | a |
| ‘Moscatel de Bago Miúdo’ | 91.25 | a |
| ‘Moscatel de Hamburgo’ | 91.25 | a |
| ‘Palomino’ | 91.25 | a |
| ‘Pedral’ | 91.25 | a |
| ‘Blanca de Monterrei’ | 90.70 | a |
| ‘Pirixileira’ | 89.23 | a |
| ‘Garnacha’ | 88.25 | a |
| ‘Picapoll Negro’ | 88.23 | a |
| ‘EVEGA 3’ | 87.97 | a |
| ‘EVEGA 6’ | 87.97 | a |
| ‘Batoca’ | 86.17 | a |
| ‘Espadeiro’ | 86.17 | a |
| ‘Sousón’ | 86.17 | a |
| ‘Tempranillo’ | 86.17 | a |
| ‘Caíño Tinto’ | 85.60 | a |
| ‘Castañal’ | 85.60 | a |
| ‘Catalán’ | 84.47 | a |
| ‘Caíño Blanco’ | 83.97 | a |
| ‘Caíño Longo 1’ | 83.97 | a |
| ‘Jarrosuelto’ | 83.97 | a |
| ‘Loureira’ | 83.97 | a |
| ‘Pan y Carne’ | 83.97 | a |
| ‘Zamarrica’ | 83.97 | a |
| ‘Dona Branca’ | 82.62 | a |
| ‘Ferrón’ | 82.62 | a |
| ‘Planta Fina’ | 82.62 | a |
| ‘Caíño Bravo’ | 81.08 | a |
| ‘Albilla do Avia’ | 80.97 | a |
| ‘Verdello Blanco’ | 76.27 | a |
| ‘Agudelo’ | 74.57 | a |
| ‘Albarín Tinto’ | 73.08 | a |
| ‘Brancellao’ | 72.72 | a |
| ‘Brancellao Blanco’ | 72.72 | a |
| ‘Caíño Longo 2’ | 72.72 | a |
| ‘Corbillón’ | 72.72 | a |
| ‘Silveiriña’ | 68.12 | a |
| ‘Mouratón’ | 67.52 | a |
| ‘Gran Negro’ | 66.13 | a |
| ‘Italia’ | 66.13 | a |
| ‘Mencía’ | 66.13 | a |
| ‘Branco Lexítimo’ | 64.17 | a |
| ‘Fernao Pires’ | 63.13 | a |
| ‘Merenzao’ | 63.13 | a |
| ‘Godello’ | 62.18 | a |

**Table S5:** Mean values and statistical differences in Growing Degrees Days (GDD) at flowering between varieties (p < 0.05).

| **Variety** | **Averages (GDD)** | **Groups** | |
| --- | --- | --- | --- |
| ‘Sousón’ | 450.08 | a |  |
| ‘Ferrón’ | 444.30 | a | b |
| ‘Caíño Longo 2’ | 434.22 | a | b |
| ‘Torrontés’ | 434.22 | a | b |
| ‘Picapoll Negro’ | 418.62 | a | b |
| ‘Caíño Longo 1’ | 418.39 | a | b |
| ‘Batoca’ | 414.60 | a | b |
| ‘Caíño Tinto’ | 414.60 | a | b |
| ‘Dona Branca’ | 414.60 | a | b |
| ‘Espadeiro’ | 414.60 | a | b |
| ‘EVEGA 4’ | 414.60 | a | b |
| ‘Garnacha’ | 414.60 | a | b |
| ‘Italia’ | 414.60 | a | b |
| ‘Mandón’ | 414.60 | a | b |
| ‘Pan y Carne’ | 414.60 | a | b |
| ‘Treixadura’ | 414.60 | a | b |
| ‘Mencía’ | 414.02 | a | b |
| ‘Albariño’ | 410.93 | a | b |
| ‘Caíño Blanco’ | 410.93 | a | b |
| ‘Gran Negro’ | 410.93 | a | b |
| ‘Loureira’ | 410.93 | a | b |
| ‘Agudelo’ | 399.00 | a | b |
| ‘Albarín Tinto’ | 399.00 | a | b |
| ‘Albilla do Avia’ | 399.00 | a | b |
| ‘Blanca de Monterrei’ | 399.00 | a | b |
| ‘Brancellao’ | 399.00 | a | b |
| ‘Brancellao Blanco’ | 399.00 | a | b |
| ‘Branco Lexítimo’ | 399.00 | a | b |
| ‘Castañal’ | 399.00 | a | b |
| ‘Caíño Bravo’ | 399.00 | a | b |
| ‘Corbillón’ | 399.00 | a | b |
| ‘EVEGA 3’ | 399.00 | a | b |
| ‘EVEGA 6’ | 399.00 | a | b |
| ‘Fernao Pires’ | 399.00 | a | b |
| ‘Garrido Fino’ | 399.00 | a | b |
| ‘Híbrido’ | 399.00 | a | b |
| ‘Jarrosuelto’ | 399.00 | a | b |
| ‘Lado’ | 399.00 | a | b |
| ‘Merenzao’ | 399.00 | a | b |
| ‘Moscatel de Bago Miúdo’ | 399.00 | a | b |
| ‘Moscatel de Hamburgo’ | 399.00 | a | b |
| ‘Mouratón’ | 399.00 | a | b |
| ‘Palomino’ | 399.00 | a | b |
| ‘Pedral’ | 399.00 | a | b |
| ‘Pirixileira’ | 399.00 | a | b |
| ‘Planta Fina’ | 399.00 | a | b |
| ‘Ratiño’ | 399.00 | a | b |
| ‘Silveiriña’ | 399.00 | a | b |
| ‘Verdello Blanco’ | 399.00 | a | b |
| ‘Zamarrica’ | 399.00 | a | b |
| ‘Godello’ | 387.03 | a | b |
| ‘Tempranillo’ | 387.03 | a | b |
| ‘Catalán’ | 364.65 |  | b |

**Table S6**: Mean values and statistical differences in Growing Degrees Days (GDD) at veraison between varieties (p < 0.05).

| **Variety** | **Average (GDD)** | **Groups** | | |
| --- | --- | --- | --- | --- |
| ‘Caíño Blanco’ | 1378.10 | a |  |  |
| ‘Agudelo’ | 1351.40 | a | b |  |
| ‘Caíño Bravo’ | 1346.93 | a | b |  |
| ‘Ferrón’ | 1346.93 | a | b |  |
| ‘Corbillón’ | 1345.58 | a | b |  |
| ‘Ratiño’ | 1345.58 | a | b |  |
| ‘Treixadura’ | 1345.58 | a | b |  |
| ‘Zamarrica’ | 1321.25 | a | b | c |
| ‘Caíño Longo 2’ | 1318.88 | a | b | c |
| ‘Garnacha’ | 1318.88 | a | b | c |
| ‘Híbrido’ | 1318.88 | a | b | c |
| ‘Loureira’ | 1318.88 | a | b | c |
| ‘Mandón’ | 1318.88 | a | b | c |
| ‘Espadeiro’ | 1314.45 | a | b | c |
| ‘Torrontés’ | 1314.45 | a | b | c |
| ‘Catalán’ | 1293.20 | a | b | c |
| ‘EVEGA 6’ | 1293.20 | a | b | c |
| ‘Jarrosuelto’ | 1293.20 | a | b | c |
| ‘Albariño’ | 1292.22 | a | b | c |
| ‘Batoca’ | 1292.22 | a | b | c |
| ‘Caíño Longo 1’ | 1290.12 | a | b | c |
| ‘Caíño Tinto’ | 1287.75 | a | b | c |
| ‘Pedral’ | 1287.75 | a | b | c |
| ‘Picapoll Negro’ | 1287.75 | a | b | c |
| ‘Sousón’ | 1265.70 | a | b | c |
| ‘Brancellao Blanco’ | 1262.07 | a | b | c |
| ‘Castañal’ | 1262.07 | a | b | c |
| ‘EVEGA 3’ | 1262.07 | a | b | c |
| ‘Garrido Fino’ | 1262.07 | a | b | c |
| ‘Italia’ | 1262.07 | a | b | c |
| ‘Verdello Blanco’ | 1262.07 | a | b | c |
| ‘Albilla do Avia’ | 1257.80 | a | b | c |
| ‘Planta Fina’ | 1235.50 | a | b | c |
| ‘Brancellao’ | 1234.57 | a | b | c |
| ‘Palomino’ | 1234.57 | a | b | c |
| ‘Silveiriña’ | 1234.57 | a | b | c |
| ‘Lado’ | 1212.33 | a | b | c |
| ‘Fernao Pires’ | 1205.55 | a | b | c |
| ‘EVEGA 4’ | 1182.38 | a | b | c |
| ‘Moscatel de Bago Miúdo’ | 1182.38 | a | b | c |
| ‘Blanca de Monterrei’ | 1182.32 | a | b | c |
| ‘Dona Branca’ | 1182.32 | a | b | c |
| ‘Pan y Carne’ | 1182.32 | a | b | c |
| ‘Moscatel de Hamburgo’ | 1171.90 | a | b | c |
| ‘Albarín Tinto’ | 1166.63 | a | b | c |
| ‘Branco Lexítimo’ | 1157.47 | a | b | c |
| ‘Tempranillo’ | 1157.47 | a | b | c |
| ‘Godello’ | 1152.37 | a | b | c |
| ‘Gran Negro’ | 1152.37 | a | b | c |
| ‘Merenzao’ | 1141.88 |  | b | c |
| ‘Mencía’ | 1137.78 |  | b | c |
| ‘Mouratón’ | 1137.78 |  | b | c |
| ‘Pirixileira’ | 1091.98 |  |  | c |

**Table S7**: Mean values and statistical differences in Growing Degrees Days (GDD) at harvest between varieties (p < 0.05).

| **Variety** | **Average (GDD)** | **Groups** | |
| --- | --- | --- | --- |
| ‘Pedral’ | 1697.30 | a |  |
| ‘Castañal’ | 1688.48 | a | b |
| ‘Caíño Bravo’ | 1688.48 | a | b |
| ‘Gran Negro’ | 1688.48 | a | b |
| ‘Jarrosuelto’ | 1688.48 | a | b |
| ‘Garrido Fino’ | 1686.57 | a | b |
| ‘Caíño Tinto’ | 1686.52 | a | b |
| ‘Espadeiro’ | 1686.52 | a | b |
| ‘Italia’ | 1684.35 | a | b |
| ‘EVEGA 4’ | 1682.62 | a | b |
| ‘Palomino’ | 1676.13 | a | b |
| ‘Ferrón’ | 1671.00 | a | b |
| ‘Sousón’ | 1671.00 | a | b |
| ‘Loureira’ | 1665.23 | a | b |
| ‘Caíño Longo 1’ | 1642.85 | a | b |
| ‘Catalán’ | 1638.57 | a | b |
| ‘Zamarrica’ | 1636.15 | a | b |
| ‘Caíño Longo 2’ | 1633.58 | a | b |
| ‘Caíño Blanco’ | 1629.28 | a | b |
| ‘Silveiriña’ | 1628.13 | a | b |
| ‘Picapoll Negro’ | 1624.40 | a | b |
| ‘Moscatel de Hamburgo’ | 1618.55 | a | b |
| ‘Torrontés’ | 1614.42 | a | b |
| ‘Treixadura’ | 1614.42 | a | b |
| ‘Brancellao Blanco’ | 1613.95 | a | b |
| ‘Batoca’ | 1608.95 | a | b |
| ‘Híbrido’ | 1605.20 | a | b |
| ‘Mandón’ | 1600.20 | a | b |
| ‘Brancellao’ | 1599.95 | a | b |
| ‘Corbillón’ | 1598.42 | a | b |
| ‘Pirixileira’ | 1592.62 | a | b |
| ‘EVEGA 6’ | 1586.02 | a | b |
| ‘Albarín Tinto’ | 1584.13 | a | b |
| ‘Ratiño’ | 1578.05 | a | b |
| ‘Agudelo’ | 1575.75 | a | b |
| ‘Mouratón’ | 1572.17 | a | b |
| ‘Merenzao’ | 1571.68 | a | b |
| ‘Albilla do Avia’ | 1570.42 | a | b |
| ‘Blanca de Monterrei’ | 1568.88 | a | b |
| ‘Garnacha’ | 1563.77 | a | b |
| ‘Lado’ | 1562.18 | a | b |
| ‘Albariño’ | 1562.02 | a | b |
| ‘Verdello Blanco’ | 1559.62 | a | b |
| ‘Fernao Pires’ | 1559.12 | a | b |
| ‘Mencía’ | 1558.43 | a | b |
| ‘Moscatel de Bago Miúdo’ | 1557.83 | a | b |
| ‘Planta Fina’ | 1550.07 | a | b |
| ‘Dona Branca’ | 1538.38 | a | b |
| ‘Pan y Carne’ | 1535.53 | a | b |
| ‘Tempranillo’ | 1535.53 | a | b |
| ‘Godello’ | 1531.18 | a | b |
| ‘EVEGA 3’ | 1491.88 | a | b |
| ‘Branco Lexítimo’ | 1463.22 |  | b |

**Table S8**: Mean values and statistical differences in Growing Degrees Days (GDD) of the period budburst-harvest between varieties (p < 0.05).

| **Variety** | **Average (GDD)** | **Groups** | |
| --- | --- | --- | --- |
| ‘Gran Negro’ | 1622.35 | a |  |
| ‘Italia’ | 1618.22 | a |  |
| ‘Caíño Bravo’ | 1607.40 | a | b |
| ‘Pedral’ | 1606.05 | a | b |
| ‘Jarrosuelto’ | 1604.52 | a | b |
| ‘Castañal’ | 1602.88 | a | b |
| ‘Caíño Tinto’ | 1600.92 | a | b |
| ‘Espadeiro’ | 1600.35 | a | b |
| ‘Garrido Fino’ | 1593.07 | a | b |
| ‘EVEGA 4’ | 1591.13 | a | b |
| ‘Ferrón’ | 1588.38 | a | b |
| ‘Palomino’ | 1584.88 | a | b |
| ‘Sousón’ | 1584.83 | a | b |
| ‘Loureira’ | 1581.27 | a | b |
| ‘Caíño Longo 2’ | 1560.87 | a | b |
| ‘Silveiriña’ | 1560.02 | a | b |
| ‘Caíño Longo 1’ | 1558.88 | a | b |
| ‘Catalán’ | 1554.10 | a | b |
| ‘Zamarrica’ | 1552.18 | a | b |
| ‘Caíño Blanco’ | 1545.32 | a | b |
| ‘Brancellao Blanco’ | 1541.23 | a | b |
| ‘Picapoll Negro’ | 1536.17 | a | b |
| ‘Moscatel de Hamburgo’ | 1527.30 | a | b |
| ‘Brancellao’ | 1527.23 | a | b |
| ‘Corbillón’ | 1525.70 | a | b |
| ‘Batoca’ | 1522.78 | a | b |
| ‘Treixadura’ | 1520.55 | a | b |
| ‘Torrontés’ | 1513.27 | a | b |
| ‘Híbrido’ | 1511.70 | a | b |
| ‘Albarín Tinto’ | 1511.05 | a | b |
| ‘Merenzao’ | 1508.55 | a | b |
| ‘Mouratón’ | 1504.65 | a | b |
| ‘Pirixileira’ | 1503.38 | a | b |
| ‘Agudelo’ | 1501.18 | a | b |
| ‘Mandón’ | 1501.07 | a | b |
| ‘EVEGA 6’ | 1498.05 | a | b |
| ‘Fernao Pires’ | 1495.98 | a | b |
| ‘Mencía’ | 1492.30 | a | b |
| ‘Albilla do Avia’ | 1489.45 | a | b |
| ‘Verdello Blanco’ | 1483.35 | a | b |
| ‘Blanca de Monterrei’ | 1478.18 | a | b |
| ‘Ratiño’ | 1476.90 | a | b |
| ‘Garnacha’ | 1475.52 | a | b |
| ‘Lado’ | 1470.93 | a | b |
| ‘Godello’ | 1469.00 | a | b |
| ‘Albariño’ | 1468.15 | a | b |
| ‘Planta Fina’ | 1467.45 | a | b |
| ‘Moscatel de Bago Miúdo’ | 1466.58 | a | b |
| ‘Dona Branca’ | 1455.77 | a | b |
| ‘Pan y Carne’ | 1451.57 | a | b |
| ‘Tempranillo’ | 1449.37 | a | b |
| ‘EVEGA 3’ | 1403.92 |  | b |
| ‘Branco Lexítimo’ | 1399.05 |  | b |

**Table S9**: Mean values and statistical differences in total acidity (g/L tartaric acid) in berry between varieties (p < 0.05).

| **Variety** | **Average** | **Groups** | | | | |
| --- | --- | --- | --- | --- | --- | --- |
| ‘Ferrón’ | 9.94 | a |  |  |  |  |
| ‘Caíño Tinto’ | 9.54 | a | b |  |  |  |
| ‘Branco Lexítimo’ | 8.13 | a | b | c |  |  |
| ‘Caíño Longo 1’ | 8.10 | a | b | c |  |  |
| ‘Caíño Longo 2’ | 8.02 | a | b | c |  |  |
| ‘Catalán’ | 7.88 | a | b | c |  |  |
| ‘Zamarrica’ | 7.78 | a | b | c | d |  |
| ‘Ratiño’ | 7.70 | a | b | c | d |  |
| ‘Loureira’ | 7.70 | a | b | c | d |  |
| ‘Albariño’ | 7.40 | a | b | c | d | e |
| ‘Caíño Bravo’ | 7.10 | a | b | c | d | e |
| ‘Caíño Blanco’ | 6.98 | a | b | c | d | e |
| ‘Lado’ | 6.87 | a | b | c | d | e |
| ‘Pedral’ | 6.85 | a | b | c | d | e |
| ‘Brancellao’ | 6.78 | a | b | c | d | e |
| ‘Picapoll Negro’ | 6.70 | a | b | c | d | e |
| ‘EVEGA 3’ | 6.62 | a | b | c | d | e |
| ‘Albarín Tinto’ | 6.60 | a | b | c | d | e |
| ‘Jarrosuelto’ | 6.50 | a | b | c | d | e |
| ‘Fernao Pires’ | 6.23 | a | b | c | d | e |
| ‘Batoca’ | 6.17 | a | b | c | d | e |
| ‘Sousón’ | 6.13 | a | b | c | d | e |
| ‘Pan y Carne’ | 5.88 | a | b | c | d | e |
| ‘Garnacha’ | 5.80 | a | b | c | d | e |
| ‘Moscatel de Bago Miúdo’ | 5.70 |  | b | c | d | e |
| ‘Verdello Blanco’ | 5.70 |  | b | c | d | e |
| ‘Godello’ | 5.63 |  | b | c | d | e |
| ‘Corbillón’ | 5.62 |  | b | c | d | e |
| ‘Brancellao Blanco’ | 5.53 |  | b | c | d | e |
| ‘Espadeiro’ | 5.44 |  | b | c | d | e |
| ‘Agudelo’ | 5.43 |  | b | c | d | e |
| ‘EVEGA 6’ | 5.30 |  |  | c | d | e |
| ‘Castañal’ | 5.28 |  |  | c | d | e |
| ‘Silveiriña’ | 5.27 |  |  | c | d | e |
| ‘Mouratón’ | 5.24 |  |  | c | d | e |
| ‘Italia’ | 5.20 |  |  | c | d | e |
| ‘Mandón’ | 5.12 |  |  | c | d | e |
| ‘Dona Branca’ | 5.07 |  |  | c | d | e |
| ‘Planta Fina’ | 5.05 |  |  | c | d | e |
| ‘EVEGA 4’ | 5.00 |  |  | c | d | e |
| ‘Albilla do Avia’ | 4.87 |  |  | c | d | e |
| ‘Garrido Fino’ | 4.85 |  |  | c | d | e |
| ‘Treixadura’ | 4.83 |  |  | c | d | e |
| ‘Moscatel de Hamburgo’ | 4.80 |  |  | c | d | e |
| ‘Híbrido’ | 4.74 |  |  | c | d | e |
| ‘Blanca de Monterrei’ | 4.50 |  |  | c | d | e |
| ‘Merenzao’ | 4.44 |  |  | c | d | e |
| ‘Tempranillo’ | 4.44 |  |  | c | d | e |
| ‘Torrontés’ | 4.40 |  |  | c | d | e |
| ‘Mencía’ | 4.36 |  |  | c | d | e |
| ‘Gran Negro’ | 4.28 |  |  | c | d | e |
| ‘Palomino’ | 3.70 |  |  |  | d | e |
| ‘Pirixileira’ | 3.40 |  |  |  |  | e |

**Table S10**: Mean values and statistical differences in Brix degree (º Brix) in berry between varieties (p < 0.05).

| **Variety** | **Average** | **Groups** | | | | | | | |
| --- | --- | --- | --- | --- | --- | --- | --- | --- | --- |
| ‘Verdello Blanco’ | 25.00 | a |  |  |  |  |  |  |  |
| ‘Pan y Carne’ | 24.84 | a | b |  |  |  |  |  |  |
| ‘Merenzao’ | 24.00 | a | b | c |  |  |  |  |  |
| ‘EVEGA 3’ | 23.76 | a | b | c |  |  |  |  |  |
| ‘Moscatel de Bago Miúdo’ | 23.47 | a | b | c | d |  |  |  |  |
| ‘Albilla do Avia’ | 23.20 | a | b | c | d | e |  |  |  |
| ‘Lado’ | 23.03 | a | b | c | d | e | f |  |  |
| ‘Tempranillo’ | 22.98 | a | b | c | d | e | f |  |  |
| ‘Treixadura’ | 22.87 | a | b | c | d | e | f |  |  |
| ‘Torrontés’ | 22.83 | a | b | c | d | e | f |  |  |
| ‘Caíño Blanco’ | 22.75 | a | b | c | d | e | f |  |  |
| ‘Branco Lexítimo’ | 22.67 | a | b | c | d | e | f | g |  |
| ‘Garnacha’ | 22.61 | a | b | c | d | e | f | g |  |
| ‘Albariño’ | 22.60 | a | b | c | d | e | f | g |  |
| ‘Híbrido’ | 22.49 | a | b | c | d | e | f | g | h |
| ‘EVEGA 6’ | 22.46 | a | b | c | d | e | f | g | h |
| ‘Caíño Longo 1’ | 22.32 | a | b | c | d | e | f | g | h |
| ‘Corbillón’ | 22.30 | a | b | c | d | e | f | g | h |
| ‘Albarín Tinto’ | 22.20 | a | b | c | d | e | f | g | h |
| ‘Moscatel de Hamburgo’ | 22.02 | a | b | c | d | e | f | g | h |
| ‘Pedral’ | 22.00 | a | b | c | d | e | f | g | h |
| ‘Godello’ | 21.97 | a | b | c | d | e | f | g | h |
| ‘Sousón’ | 21.95 | a | b | c | d | e | f | g | h |
| ‘Castañal’ | 21.88 | a | b | c | d | e | f | g | h |
| ‘EVEGA 4’ | 21.62 | a | b | c | d | e | f | g | h |
| ‘Picapoll Negro’ | 21.60 | a | b | c | d | e | f | g | h |
| ‘Mencía’ | 21.55 | a | b | c | d | e | f | g | h |
| ‘Brancellao’ | 21.55 | a | b | c | d | e | f | g | h |
| ‘Caíño Longo 2’ | 21.52 | a | b | c | d | e | f | g | h |
| ‘Mouratón’ | 21.50 | a | b | c | d | e | f | g | h |
| ‘Ratiño’ | 21.43 | a | b | c | d | e | f | g | h |
| ‘Mandón’ | 21.42 | a | b | c | d | e | f | g | h |
| ‘Espadeiro’ | 21.38 | a | b | c | d | e | f | g | h |
| ‘Dona Branca’ | 21.33 | a | b | c | d | e | f | g | h |
| ‘Planta Fina’ | 21.30 | a | b | c | d | e | f | g | h |
| ‘Ferrón’ | 21.06 | a | b | c | d | e | f | g | h |
| ‘Fernao Pires’ | 20.90 |  | b | c | d | e | f | g | h |
| ‘Italia’ | 20.85 |  |  | c | d | e | f | g | h |
| ‘Caíño Bravo’ | 20.72 |  |  | c | d | e | f | g | h |
| ‘Batoca’ | 20.67 |  |  | c | d | e | f | g | h |
| ‘Agudelo’ | 20.50 |  |  | c | d | e | f | g | h |
| ‘Catalán’ | 20.26 |  |  | c | d | e | f | g | h |
| ‘Zamarrica’ | 20.20 |  |  | c | d | e | f | g | h |
| ‘Garrido Fino’ | 20.10 |  |  | c | d | e | f | g | h |
| ‘Jarrosuelto’ | 19.75 |  |  |  | d | e | f | g | h |
| ‘Palomino’ | 19.73 |  |  |  | d | e | f | g | h |
| ‘Caíño Tinto’ | 19.66 |  |  |  | d | e | f | g | h |
| ‘Pirixileira’ | 19.33 |  |  |  |  | e | f | g | h |
| ‘Loureira’ | 19.23 |  |  |  |  |  | f | g | h |
| ‘Silveiriña’ | 19.20 |  |  |  |  |  | f | g | h |
| ‘Gran Negro’ | 19.13 |  |  |  |  |  | f | g | h |
| ‘Brancellao Blanco’ | 18.73 |  |  |  |  |  |  | g | h |
| ‘Blanca de Monterrei’ | 18.63 |  |  |  |  |  |  |  | h |

**Table S11**: Mean values and statistical differences in pH in berry between varieties (p < 0.05).

| **Variety** | **Average** | **Groups** |
| --- | --- | --- |
| ‘Pirixileira’ | 3.71 | a |
| ‘Treixadura’ | 3.69 | a |
| ‘Tempranillo’ | 3.68 | a |
| ‘Gran Negro’ | 3.68 | a |
| ‘Mencía’ | 3.67 | a |
| ‘Torrontés’ | 3.66 | a |
| ‘Palomino’ | 3.62 | a |
| ‘Italia’ | 3.62 | a |
| ‘EVEGA 4’ | 3.61 | a |
| ‘Moscatel de Hamburgo’ | 3.60 | a |
| ‘Planta Fina’ | 3.60 | a |
| ‘Mouratón’ | 3.59 | a |
| ‘EVEGA 6’ | 3.58 | a |
| ‘Dona Branca’ | 3.58 | a |
| ‘Pan y Carne’ | 3.57 | a |
| ‘Castañal’ | 3.55 | a |
| ‘Espadeiro’ | 3.55 | a |
| ‘Merenzao’ | 3.54 | a |
| ‘Corbillón’ | 3.54 | a |
| ‘Blanca de Monterrei’ | 3.52 | a |
| ‘Caíño Bravo’ | 3.51 | a |
| ‘Brancellao Blanco’ | 3.50 | a |
| ‘Godello’ | 3.50 | a |
| ‘Híbrido’ | 3.50 | a |
| ‘Sousón’ | 3.49 | a |
| ‘Agudelo’ | 3.48 | a |
| ‘Moscatel de Bago Miúdo’ | 3.48 | a |
| ‘Garrido Fino’ | 3.48 | a |
| ‘Mandón’ | 3.47 | a |
| ‘Silveiriña’ | 3.46 | a |
| ‘Albilla do Avia’ | 3.45 | a |
| ‘Verdello Blanco’ | 3.45 | a |
| ‘Batoca’ | 3.42 | a |
| ‘Fernao Pires’ | 3.42 | a |
| ‘EVEGA 3’ | 3.36 | a |
| ‘Jarrosuelto’ | 3.36 | a |
| ‘Caíño Blanco’ | 3.35 | a |
| ‘Ratiño’ | 3.33 | a |
| ‘Albarín Tinto’ | 3.30 | a |
| ‘Lado’ | 3.30 | a |
| ‘Caíño Longo 2’ | 3.29 | a |
| ‘Picapoll Negro’ | 3.29 | a |
| ‘Albariño’ | 3.29 | a |
| ‘Caíño Longo 1’ | 3.28 | a |
| ‘Garnacha’ | 3.26 | a |
| ‘Catalán’ | 3.26 | a |
| ‘Caíño Tinto’ | 3.26 | a |
| ‘Brancellao’ | 3.26 | a |
| ‘Pedral’ | 3.24 | a |
| ‘Zamarrica’ | 3.22 | a |
| ‘Branco Lexítimo’ | 3.19 | a |
| ‘Ferrón’ | 3.13 | a |
| ‘Loureira’ | 3.12 | a |

**Table S12:** Mean values and statistical differences in tartaric acid (g/L) in berry between varieties (p < 0.05).

| **Variety** | **Average** | **Groups** | | | |
| --- | --- | --- | --- | --- | --- |
| ‘Zamarrica’ | 7.48 | a |  |  |  |
| ‘Loureira’ | 7.45 | a | b |  |  |
| ‘Branco Lexítimo’ | 7.30 | a | b | c |  |
| ‘Verdello Blanco’ | 7.07 | a | b | c | d |
| ‘Planta Fina’ | 7.00 | a | b | c | d |
| ‘Albariño’ | 6.97 | a | b | c | d |
| ‘Brancellao’ | 6.88 | a | b | c | d |
| ‘Ratiño’ | 6.80 | a | b | c | d |
| ‘Catalán’ | 6.80 | a | b | c | d |
| ‘Garnacha’ | 6.68 | a | b | c | d |
| ‘Lado’ | 6.67 | a | b | c | d |
| ‘Godello’ | 6.60 | a | b | c | d |
| ‘Mandón’ | 6.54 | a | b | c | d |
| ‘Picapoll Negro’ | 6.54 | a | b | c | d |
| ‘Albilla do Avia’ | 6.53 | a | b | c | d |
| ‘Caíño Blanco’ | 6.45 | a | b | c | d |
| ‘Caíño Longo 1’ | 6.36 | a | b | c | d |
| ‘Torrontés’ | 6.27 | a | b | c | d |
| ‘Silveiriña’ | 6.27 | a | b | c | d |
| ‘EVEGA 3’ | 6.18 | a | b | c | d |
| ‘Albarín Tinto’ | 6.16 | a | b | c | d |
| ‘Pedral’ | 6.15 | a | b | c | d |
| ‘Brancellao Blanco’ | 6.13 | a | b | c | d |
| ‘Ferrón’ | 6.08 | a | b | c | d |
| ‘Garrido Fino’ | 6.05 | a | b | c | d |
| ‘Batoca’ | 6.03 | a | b | c | d |
| ‘Corbillón’ | 5.94 | a | b | c | d |
| ‘Híbrido’ | 5.92 | a | b | c | d |
| ‘EVEGA 6’ | 5.88 | a | b | c | d |
| ‘Fernao Pires’ | 5.87 | a | b | c | d |
| ‘Moscatel de Bago Miúdo’ | 5.83 | a | b | c | d |
| ‘Merenzao’ | 5.68 | a | b | c | d |
| ‘Pan y Carne’ | 5.68 | a | b | c | d |
| ‘Italia’ | 5.65 | a | b | c | d |
| ‘Palomino’ | 5.60 | a | b | c | d |
| ‘Caíño Longo 2’ | 5.58 | a | b | c | d |
| ‘Jarrosuelto’ | 5.50 | a | b | c | d |
| ‘Caíño Tinto’ | 5.48 | a | b | c | d |
| ‘Tempranillo’ | 5.42 | a | b | c | d |
| ‘Dona Branca’ | 5.37 | a | b | c | d |
| ‘Treixadura’ | 5.33 | a | b | c | d |
| ‘Mencía’ | 5.28 | a | b | c | d |
| ‘Pirixileira’ | 5.20 | a | b | c | d |
| ‘Agudelo’ | 5.13 | a | b | c | d |
| ‘Moscatel de Hamburgo’ | 5.00 | a | b | c | d |
| ‘Blanca de Monterrei’ | 4.90 | a | b | c | d |
| ‘EVEGA 4’ | 4.84 | a | b | c | d |
| ‘Gran Negro’ | 4.75 |  | b | c | d |
| ‘Mouratón’ | 4.74 |  | b | c | d |
| ‘Castañal’ | 4.68 |  |  | c | d |
| ‘Caíño Bravo’ | 4.68 |  |  | c | d |
| ‘Espadeiro’ | 4.58 |  |  | c | d |
| ‘Sousón’ | 4.40 |  |  |  | d |

**Table S13:** Mean values and statistical differences in malic acid (g/L) in berry between varieties (p < 0.05).

| **Variety** | **Average** | **Groups** | | | | | | | |
| --- | --- | --- | --- | --- | --- | --- | --- | --- | --- |
| ‘Caíño Tinto’ | 7.04 | a |  |  |  |  |  |  |  |
| ‘Ferrón’ | 6.98 | a | b |  |  |  |  |  |  |
| ‘Caíño Longo 1’ | 5.44 | a | b | c |  |  |  |  |  |
| ‘Caíño Longo 2’ | 5.26 | a | b | c |  |  |  |  |  |
| ‘Caíño Bravo’ | 5.20 | a | b | c | d |  |  |  |  |
| ‘Albariño’ | 5.13 | a | b | c | d | e |  |  |  |
| ‘Ratiño’ | 4.67 | a | b | c | d | e | f |  |  |
| ‘Caíño Blanco’ | 3.93 | a | b | c | d | e | f | g |  |
| ‘Jarrosuelto’ | 3.90 | a | b | c | d | e | f | g |  |
| ‘Sousón’ | 3.78 |  | b | c | d | e | f | g |  |
| ‘Pan y Carne’ | 3.56 |  |  | c | d | e | f | g | h |
| ‘Mouratón’ | 3.48 |  |  | c | d | e | f | g | h |
| ‘Pedral’ | 3.33 |  |  | c | d | e | f | g | h |
| ‘Fernao Pires’ | 3.30 |  |  | c | d | e | f | g | h |
| ‘Espadeiro’ | 3.28 |  |  | c | d | e | f | g | h |
| ‘Albarín Tinto’ | 3.24 |  |  | c | d | e | f | g | h |
| ‘EVEGA 3’ | 3.22 |  |  | c | d | e | f | g | h |
| ‘Catalán’ | 3.16 |  |  | c | d | e | f | g | h |
| ‘Batoca’ | 3.10 |  |  | c | d | e | f | g | h |
| ‘Branco Lexítimo’ | 3.10 |  |  | c | d | e | f | g | h |
| ‘Lado’ | 3.07 |  |  | c | d | e | f | g | h |
| ‘Italia’ | 3.05 |  |  | c | d | e | f | g | h |
| ‘Picapoll Negro’ | 3.04 |  |  | c | d | e | f | g | h |
| ‘Loureira’ | 3.03 |  |  | c | d | e | f | g | h |
| ‘Brancellao’ | 3.00 |  |  | c | d | e | f | g | h |
| ‘EVEGA 6’ | 3.00 |  |  | c | d | e | f | g | h |
| ‘Agudelo’ | 2.97 |  |  | c | d | e | f | g | h |
| ‘Dona Branca’ | 2.93 |  |  | c | d | e | f | g | h |
| ‘Zamarrica’ | 2.88 |  |  | c | d | e | f | g | h |
| ‘Moscatel de Bago Miúdo’ | 2.87 |  |  | c | d | e | f | g | h |
| ‘Corbillón’ | 2.82 |  |  | c | d | e | f | g | h |
| ‘EVEGA 4’ | 2.82 |  |  | c | d | e | f | g | h |
| ‘Treixadura’ | 2.80 |  |  | c | d | e | f | g | h |
| ‘Castañal’ | 2.78 |  |  | c | d | e | f | g | h |
| ‘Moscatel de Hamburgo’ | 2.72 |  |  | c | d | e | f | g | h |
| ‘Tempranillo’ | 2.50 |  |  | c | d | e | f | g | h |
| ‘Mencía’ | 2.44 |  |  | c | d | e | f | g | h |
| ‘Godello’ | 2.33 |  |  | c | d | e | f | g | h |
| ‘Planta Fina’ | 2.20 |  |  | c | d | e | f | g | h |
| ‘Verdello Blanco’ | 2.20 |  |  | c | d | e | f | g | h |
| ‘Brancellao Blanco’ | 1.97 |  |  |  | d | e | f | g | h |
| ‘Silveiriña’ | 1.90 |  |  |  |  | e | f | g | h |
| ‘Blanca de Monterrei’ | 1.90 |  |  |  |  | e | f | g | h |
| ‘Híbrido’ | 1.80 |  |  |  |  |  | f | g | h |
| ‘Gran Negro’ | 1.75 |  |  |  |  |  | f | g | h |
| ‘Merenzao’ | 1.62 |  |  |  |  |  | f | g | h |
| ‘Torrontés’ | 1.53 |  |  |  |  |  | f | g | h |
| ‘Garnacha’ | 1.50 |  |  |  |  |  | f | g | h |
| ‘Garrido Fino’ | 1.40 |  |  |  |  |  |  | g | h |
| ‘Mandón’ | 1.36 |  |  |  |  |  |  | g | h |
| ‘Albilla do Avia’ | 0.97 |  |  |  |  |  |  | g | h |
| ‘Pirixileira’ | 0.83 |  |  |  |  |  |  | g | h |
| ‘Palomino’ | 0.33 |  |  |  |  |  |  |  | h |

**Table S14**: Correlation coefficients of the PCA between the three first axes and the varieties for the qualitative and quantitative parameters

| **Variety** | **RPP SSR [16]** | **RRP (SNP) K2** | **Qualitative parameters** | | | **Quantitative parameters** | | |
| --- | --- | --- | --- | --- | --- | --- | --- | --- |
|  |  |  | **FAC1_1** | **FAC2_1** | **FAC3_1** | **FAC1_2** | **FAC2_2** | **FAC3_2** |
| ‘Agudelo’ | RPP1b | RPP1 | -0.59062 | -0.43284 | -0.37777 | -0.08479 | 0.27539 | -0.40420 |
| ‘Albarín Tinto’ | RPP1b | Admixed | -0.37609 | -1.01061 | -0.33867 | 0.17692 | -0.33037 | -1.04646 |
| ‘Albariño’ | RPP1a | RPP1 | -2.15155 | 2.09865 | -0.64495 | 1.19957 | 1.12533 | -0.25652 |
| ‘Albilla do Avia’ | RPP1b | RPP2 | 0.43101 | 0.16622 | -1.17105 | 1.14818 | -0.42645 | -0.20945 |
| ‘Batoca’ | RPP1b | Admixed | -0.15387 | -0.50633 | -0.94137 | -0.30496 | 0.38700 | -0.06285 |
| ‘Blanca de Monterrei’ | Admixed | RPP2 | -0.32302 | -1.45653 | -0.43509 | -1.08919 | -1.14780 | -0.80017 |
| ‘Brancellao Blanco’ | RPP1b | RPP1 | 1.78581 | 2.50903 | 1.06319 | -1.20599 | -0.26272 | -1.20889 |
| ‘Brancellao’ | RPP1b | RPP1 | 2.01311 | 2.67994 | 0.76910 | 0.10179 | 0.29124 | -1.18307 |
| ‘Branco Lexítimo’ | RPP1b | Admixed | -0.86217 | -0.75999 | -0.68969 | 1.52924 | 0.06493 | -2.75725 |
| ‘Caíño Blanco’ | RPP1a | RPP1 | -2.55467 | 2.76575 | -1.07161 | 0.61063 | 1.28872 | 0.34762 |
| ‘Caíño Bravo’ | RPP1a | RPP1 | -0.13303 | 0.01514 | 0.38321 | -1.09979 | 0.85960 | 0.94639 |
| ‘Caíño Longo 1’ | RPP1a | RPP1 | 0.07163 | -0.15454 | 0.28289 | -0.01155 | 2.35051 | 1.32658 |
| ‘Caíño Longo 2’ | RPP1a | RPP1 | 1.15189 | 0.86604 | -3.47926 | -0.31865 | 1.29137 | 0.38711 |
| ‘Caíño Tinto’ | RPP1a | RPP1 | -0.23525 | 0.38295 | 0.48090 | -1.54601 | 1.60669 | -0.20396 |
| ‘Castañal’ | RPP1a | RPP1 | 0.37547 | 0.08152 | -0.39557 | -0.72902 | -0.38112 | 1.31035 |
| ‘Catalán’ |  | RPP1 | 1.09253 | 1.10375 | -1.75217 | -0.59377 | 0.83557 | -1.65822 |
| ‘Corbillón’ | RPP1b | RPP1 | -0.46452 | -0.30437 | 0.16178 | 0.46886 | 0.34288 | 0.21941 |
| ‘Dona Branca’ | RPP2a | RPP2 | 0.12438 | -0.77058 | -1.37660 | 0.35379 | -0.88296 | 0.11604 |
| ‘Espadeiro’ | RPP1a | RPP1 | -0.98348 | -0.44627 | -0.14740 | -0.83807 | 0.16737 | 1.66724 |
| ‘EVEGA 3’ | RPP1b | Admixed | -0.61144 | -0.76967 | 0.46655 | 2.02811 | 0.30885 | -0.04379 |
| ‘EVEGA 4’ | RPP1b | Admixed | -0.35462 | -0.01921 | 0.11441 | -0.94965 | -0.97295 | 1.38655 |
| ‘EVEGA 6’ | RPP1b | Admixed | 0.74762 | -0.26235 | 0.01555 | 0.67565 | -0.06066 | 0.54964 |
| ‘Fernao Pires’ | RPP1b | RPP2 | -0.37013 | -0.71257 | 0.06751 | -0.05792 | -0.36172 | -1.23457 |
| ‘Ferrón’ | RPP1a | RPP1 | 1.23452 | 0.86075 | -0.55973 | -0.71294 | 2.43395 | 0.54101 |
| ‘Garnacha’ | RPP2a | RPP2 | 0.54012 | -0.77352 | 0.06844 | 1.12594 | 0.53339 | -0.30111 |
| ‘Garrido Fino’ | RPP1b | RPP2 | 1.13252 | -0.04711 | -0.17906 | -1.13941 | -0.32236 | 0.17443 |
| ‘Godello’ | RPP1b | RPP2 | -0.54539 | -0.40168 | -0.03088 | 0.68410 | -1.04235 | -1.58310 |
| ‘Gran Negro’ | Admixed | RPP2 | 1.80436 | 0.43965 | -0.02989 | -2.17387 | -1.62290 | 0.09041 |
| ‘Híbrido’ | RPP2b | Admixed | -0.03091 | 0.10612 | 0.36525 | 0.58656 | 0.03014 | 0.76113 |
| ‘Italia’ |  | RPP1 | 2.16324 | 0.00464 | -0.92578 | -1.08356 | -0.34453 | 0.55026 |
| ‘Jarrosuelto’ | RPP2a | RPP2 | 1.09948 | 0.00868 | 1.57926 | -1.43277 | 0.48930 | -0.15896 |
| ‘Lado’ | RPP1b | Admixed | -1.18482 | 0.19189 | 0.52162 | 1.19751 | 0.09843 | -0.40340 |
| ‘Loureira’ | RPP1a | RPP1 | -0.59851 | -0.48437 | 0.20216 | -1.12191 | 1.33974 | -1.63772 |
| ‘Mandón’ | RPP2a | RPP2 | 0.34655 | -0.94726 | 0.27860 | 0.34941 | 0.23224 | 0.33686 |
| ‘Mencía’ | RPP1b | Admixed | 0.37066 | -0.48742 | 0.42950 | 0.10840 | -1.49746 | -0.03529 |
| ‘Merenzao’ | RPP1b | Admixed | 0.02330 | -0.25835 | 0.78616 | 0.93038 | -1.45582 | 0.21334 |
| ‘Moscatel de Bago Miúdo’ | RPP2b | RPP1 | -0.35220 | -1.01782 | 0.73507 | 1.21440 | -0.65968 | 0.32725 |
| ‘Moscatel de Hamburgo’ | RPP2b | Admixed | -0.00183 | -0.87902 | 0.06321 | -0.19280 | -1.08320 | 0.82680 |
| ‘Mouratón’ | Admixed | RPP2 | 0.06501 | -1.02663 | -0.17726 | -0.12970 | -1.24216 | -0.23209 |
| ‘Palomino’ | RPP2a | RPP2 | 0.11988 | -0.56150 | -0.14052 | -1.30425 | -1.10981 | 0.29360 |
| ‘Pan y Carne’ |  | Admixed | -0.11231 | -0.81284 | 0.20055 | 1.80788 | -0.64522 | 0.99692 |
| ‘Pedral’ | RPP1b | Admixed | 0.88641 | 1.24086 | -0.54406 | -0.51282 | 0.71031 | 0.17964 |
| ‘Picapoll Negro’ |  | Admixed | -0.47435 | -0.53007 | 0.00267 | -0.03512 | 0.70027 | -0.16052 |
| ‘Pirixileira’ | RPP1a | RPP1 | 0.11786 | 0.91846 | 3.95846 | -1.05391 | -2.27256 | -0.44467 |
| ‘Planta Fina’ | RPP2a | RPP2 | 0.03904 | -1.01209 | -0.42069 | 0.65727 | -0.47773 | -0.62825 |
| ‘Ratiño’ | RPP1b | RPP1 | -2.76227 | 2.18972 | -0.38865 | 0.68197 | 1.43545 | -0.29762 |
| ‘Silveiriña’ | RPP1b | Admixed | -1.12623 | 0.07883 | 0.52394 | -1.19866 | -0.45514 | -1.24701 |
| ‘Sousón’ | RPP1a | RPP1 | 0.64181 | 0.29464 | 0.24548 | -0.65834 | 0.08899 | 1.96934 |
| ‘Tempranillo’ | RPP2a | RPP2 | 0.40358 | -0.42975 | 0.85537 | 1.06710 | -1.44436 | 0.36087 |
| ‘Torrontés’ | RPP2a | RPP2 | -0.24517 | -0.99212 | -0.58328 | 0.70853 | -0.06331 | 1.85491 |
| ‘Treixadura’ | RPP1b | Admixed | 0.09075 | -1.11255 | -0.34990 | 0.65606 | 0.12056 | 1.98631 |
| ‘Verdello Blanco’ | RPP1b | Admixed | -1.27968 | 0.22866 | 0.60283 | 2.03159 | -0.04342 | 0.21092 |
| ‘Zamarrica’ | RPP1b | RPP1 | 0.00559 | 0.14809 | 1.92725 | -0.52045 | 1.20050 | -1.73183 |


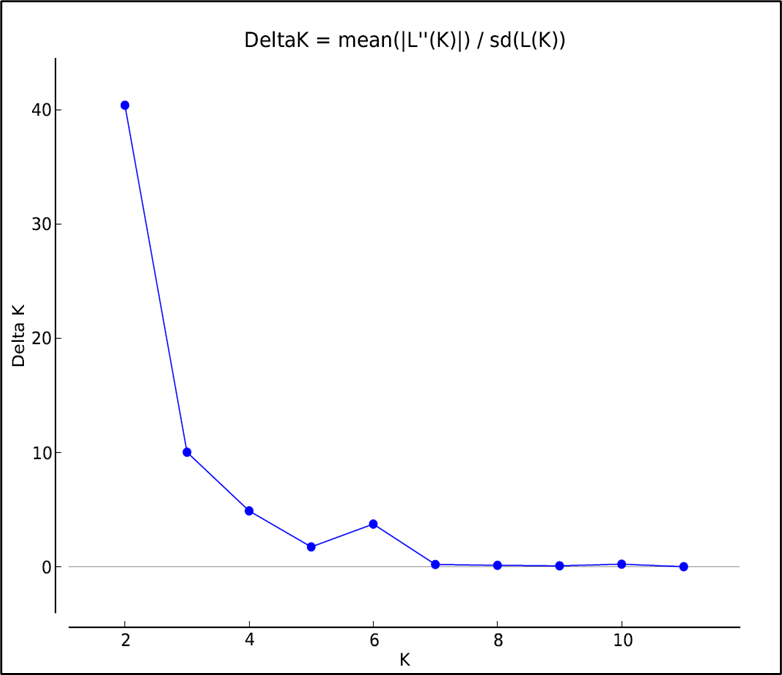


**Fig. S1:** DeltaK resulting of a Bayesian analysis with the Structure software (Pritchard et al. 2000a,b) on 48 SNPs applied on grapevines.
